# Role of memantine in adult migraine: a systematic review and network meta-analysis to compare memantine with existing migraine preventive medications

**DOI:** 10.3389/fphar.2024.1496621

**Published:** 2024-12-18

**Authors:** Guanglu Li, Baoquan Qu, Tao Zheng, Shaojie Duan, Lei Liu, Zunjing Liu

**Affiliations:** ^1^ Beijing University of Chinese Medicine, Beijing, China; ^2^ Department of Neurology, China-Japan Friendship Hospital, Beijing, China; ^3^ Department of Geriatrics, Taizhou Central Hospital (Taizhou University Hospital), Taizhou, China; ^4^ Department of Neurology, Peking University People’s Hospital, Beijing, China

**Keywords:** migraine, memantine, prevention, network meta-analysis, systematic review

## Abstract

**Background:**

While memantine has been considered a promising drug for migraine prevention, no conclusive evidence exists comparing its efficacy with other migraine-preventive medications. This network meta-analysis (NMA) aimed to access the effectiveness and acceptability of memantine and other guideline-recommended prophylactic agents for migraine.

**Methods:**

We searched the Cochrane Register of Controlled Trials, Embase, PubMed, and ClinicalTrials databases from their inception to 1 June 2024. Randomized placebo-controlled trials (RCTs) examining the pharmacological prevention of adult migraine patients were included. The primary efficacy outcome was the change in migraine days, and the primary safety outcome was withdrawal due to adverse events. Secondary outcomes included 50% response rates and frequency of any adverse events. The analysis followed the Preferred Reporting Items for Systematic Reviews and Meta-Analyses (PRISMA) guidelines.

**Results:**

Thirty-eight RCTs, including a total of 13,223 participants, were analyzed. Our analysis showed that memantine demonstrated the second-largest reduction in migraine days [standardized mean difference (SMD): −0.83; 95% confidence interval (CI): −1.26, −0.41 compared with placebo] and the highest 50% response rates [odds ratio (OR): 5.58, 95% CI: 1.31 to 23.69] in all studied interventions. Moreover, among all interventions, memantine appeared to show the lowest dropout rate and moderate frequency of adverse events. However, its confidence intervals contained null values.

**Conclusion:**

This study provides prioritisation evidence for memantine in migraine prevention, as memantine can significantly decrease the frequency of migraine attacks, improves response rates, and fair acceptability. These beneficial effects were not inferior to currently recommended pharmacological regimens. However, due to the lack of long-term efficacy and safety data, as well as few direct comparisons with active control agents, the estimates of memantine may be overly optimistic. Clinicians should interpret the findings of current NMA cautiously and apply them in a relatively conservative manner.

## Introduction

Migraine is the second most prevalent neurological disorder neurologic disorder, affecting 12%–20% of the global population (Buse et al., 2010). It is the leading cause of disability-adjusted life years (DALYs) and years lived with disability (YLD) in young women (Steiner et al., 2020; GBD 2016 Neurology Collaborators, 2019). According to Global Burden of Disease (GBD) modeling, approximately 1.04 billion people worldwide suffer from migraines (GBD 2015 Neurological Disorders Collaborator Group, 2017). The headache-related disability, financial stress, and disruption of family and social roles associated with migraine impose a severe personal burden on patients and increase the risk of progression to medication overuse headaches (Ashina et al., 2023).

Preventive treatment is an important part of migraine overall management, aimed at reducing attacks of frequency, severity, duration, and disability as well as improving responsiveness to acute treatment (Pellesi et al., 2024). Evidence-based guidelines recommend multiple preventive medications (Burch et al., 2022; Demarquay et al., 2021), including antihypertensive agents, anticonvulsants, and antidepressants. Since none of these agents are specifically designed for migraine, causing these treatments often have limited to moderate efficacy, moderate to high rates of adverse events, contraindications, or interactions that limit use (Powers et al., 2017). The new specific preventive agent, calcitonin gene-related peptide monoclonal antibodies (CGRP-mAbs), significantly reduced the number of migraine days compared to placebo and was well tolerated, but the response rates appeared modest. Only approximately one-third of the patients responded very well, and one-third do not respond at all (Tseng et al., 2024; Ferrari et al., 2022). Moreover, newer drugs, like CGRP-mAbs and gepants (Baraldi et al., 2024), are not yet available in most countries or at a much higher cost, restricting access to their use. Thus, the demand for more effective and highly acceptable agents for the prevention of migraine may remain unfulfilled.

Glutamate, one of the most abundant excitatory neurotransmitters in the central nervous system (Welch et al., 1990), is significantly elevated in the cerebrospinal fluid of migraine patients and is closely associated with migraine attacks (Vieira et al., 2007; Mistry et al., 2021). Memantine is an activity-dependent antagonist of the N-methyl-d-aspartate (NMDA) subtype of the glutamate receptor, which can reduce neuronal hyperexcitability by inhibiting the NMDA receptor (Charles, 2021). Approved by the Food and Drug Administration (FDA) for Alzheimer’s disease due to its excellent safety and tolerability, memantine also shows promise as an adjunctive treatment for conditions like schizophrenia and phantom limb pain (Loy et al., 2016). This profile suggests that memantine is theoretically and precedentially suitable as a prophylactic agent for migraine prevention.

Several studies have evaluated the efficacy and safety of memantine for migraine prevention, including two prospective open-label studies (Assarzadegan and Sistanizad, 2017; Bigal et al., 2008), two placebo-controlled RCTs (Noruzzadeh et al., 2016; Shanmugam et al., 2019), two active-controlled RCTs (Vazquez-Guevara et al., 2023; Ghorbani et al., 2015). All studies showed a reduction in migraine days and headache severity in migraine patients treated with memantine; adverse effects were rare, and none were serious. These studies establish initial evidence for the hypothesised benefits of memantine for migraine prevention. However, its relative effectiveness and safety compared to guideline-recommended medications, including CGRP-mAbs, remain uncertain. In the absence of direct comparisons, network meta-analysis (NMA) provides a method to perform multiple comparisons simultaneously in a single analysis as evidence for clinical practice. The primary objective of this study was to systematically assess and rank the effectiveness, safety, and acceptability of memantine compared to other migraine preventive agents using NMA.

## Methods

### General guidelines applied

This systematic review and meta-analysis followed the recommendations of the Preferred Reporting Items for Systematic Reviews and Meta-analyses (PRISMA) reporting guideline checklist (Supplementary Material: Appendix S1). The current study was registered in the International Prospective Register of Systematic Reviews (PROSPERO: CRD42024582523).

### Literature search

This systematic review and meta-analysis identified preventive medications for the treatment of patients with chronic or episodic migraine with or without aura. We searched the Cochrane Register of Controlled Trials, MEDLINE via PubMed, Embase and ClinicalTrials.gov, with language restrictions in English, from inception until 1 June 2024, to search for randomized trials of pharmacologic treatments for migraine prevention. We applied a combination of keywords and text words related to migraine and drugs for migraine prophylaxis. Then, we combined them with validated screening tools recommended by the Harvard Countway Library for randomized controlled clinical trials. Each database uses a specific search strategy, which can be found in Supplementary Material in Appendix S2. We additionally conducted manual searches for potentially eligible articles from existing pairwise meta-analyses and the reference lists of review articles to complement our further trials. Two authors operated the literature search process independently.

### Inclusion and exclusion criteria

To increase the reliability of the current NMA, studies needed to meet the following PICO criteria and have been peer-reviewed and formally published.

#### Participants

Participants were required to be diagnosed with episodic and chronic migraine patients (with or without aura) for 18 years and older based on the International Classification of Headache Disorders criteria (ICHD) system or the ICHD operating at the time of the study.

#### Intervention

Memantine or FDA-approval/guideline-recommendation drugs for migraine prevention. For drugs with multiple doses for administration, we included the guideline-recommended moderate dose to limit the number of intervention arms. Referring to previous studies (Herd et al., 2019; Lampl et al., 2023), we did not include botulinum toxin in our analyses.

#### Comparison

We included only randomized controlled trials (RCTs) that compared at least one pharmacologic agent with a placebo or that performed direct comparisons of at least two pharmacologic agents but applied a placebo in the study design (In clinical trials of headache and pain management, placebo effects were found to be as high as 40%–55%).

#### Outcome

The study had to report at least one clinical outcome indicator we were concerned about. We followed the International Headache Society (IHS) recommendations (Diener et al., 2020) and chose the change from baseline in migraine days per month as our primary efficacy outcome. Secondary efficacy endpoints were the response rate, defined as a 50% reduction in baseline frequency of migraine days after pharmacological interventions. The primary outcomes for the safety profile were assessed according to withdrawal due to adverse events. The secondary safety outcome was the frequency of any adverse events within post-dose.

The exclusion criteria were as follows: (1) no clinical trials in humans; (2) no inclusion of adult participants with migraine; (3) studies not published in peer-reviewed journals or studies that were *post hoc* or secondary analyses; (4) crossover studies, excluded except when the results of the first phase were given separately; (5) no use of placebo; and (6) studies involving cluster, tension, menstrual migraine or episodic and chronic migraine associated with other neurological disorders.

### Study screening and data collection

After the initial search, one pair of authors (GL Li, BQ Qu) independently performed the following operations: removing duplicates, reviewing the titles and abstracts of all identified citations for primary screening, and retrieving and screening full-text papers according to eligibility criteria. Disagreements were resolved through discussion and, if necessary, by a third senior author (L Liu). For each included study based on eligibility criteria, two authors (GL Li, BQ Qu) independently performed the data extraction with a predesigned Excel spreadsheet, which included the study title, first author name, publication year, participants’ characteristics, diagnostic criteria, sample size, intervention characteristics (dose, frequency, administration route, duration of intervention), efficacy and safety outcomes data, and information for the assessment of the risk of bias. A third author (L Liu) checked the consistency and accuracy of all extracted data. Any discrepancies in evaluating these data were resolved by discussion or consultation. A third author (L Liu) would evaluate those data that could not be determined until a consensus was reached.

### Assessment of risk of bias and quality of evidence

Two independent authors assessed each included study using a standardized table according to the modified Cochrane Risk of Bias version 2 (RoB2) tool for RCTs (Sterne et al., 2019). According to the five domains, the risk of bias for each trial was rated as low risk of bias, some concerns, and high risk of bias. Confidence in Network Meta-Analysis (CINeMA) (Nikolakopoulou et al., 2020) is an approach for determining confidence in the results of an NMA broadly based on the Grading of Recommendations Assessment, Development, and Evaluation (GRADE), with several conceptual and semantic differences. We used a freely available, user-friendly online CINeMA web application (Papakonstantinou et al., 2020) to evaluate confidence. Finally, comparative-adjusted funnel plots and Egger regression were used to assess potential minor study effects and publication bias.

### Statistical analysis

We performed NMA analyses using R (version 3.2.2) to combine direct and indirect evidence. Continuous data were calculated using summary standardised mean differences (SMDs) with 95% confidence intervals (CIs). We estimated the odds ratio (OR) with 95% CIs for categorical data.

We used the frequentist theory model with the mvmeta command to compare the effect size among studies with the same treatments. All comparisons were two-tailed, and a p-value cut-off point 0.05 denoted statistical significance. Heterogeneity among the included studies was evaluated using the tau value and the heterogeneity statistic I^2^. The Tau value is the estimated standard deviation of the effect across the included studies (Higgins et al., 2003). I^2^ values of less than 50% indicate that the heterogeneity may not be significant; a value higher than 50% may represent substantial heterogeneity. NMA relies on the transitivity assumption to estimate the effects of indirect treatment. We conducted a statistical evaluation of inconsistency. A loop-specific approach and the node-splitting method (Dias et al., 2010) were used to assess the potential local inconsistency of the model. The design-by-treatment model was applied to evaluate global inconsistency among the whole NMA. In addition, We calculated the surface under the cumulative ranking curve (SUCRA) to increase the clinical application of our study. In brief, SUCRA is one summarisation ranking metric that Salanti et al. proposed (Salanti et al., 2011). The advantage of SUCRA over the mean rank is that it has a common range from 0 to 1, facilitating consistent interpretation of different NMAs (Rosenberger et al., 2021). SUCRA values of 1 indicate that the treatment might be the best and 0 the worst.

In the sensitivity analyses, We excluded moderate to high risk studies to test whether individual studies had a significant effect on overall results. We performed one subgroup analysis: only oral agents; in other words, comparisons were performed after excluding CGRP-mAbs.

## Results

### Search results and study characteristics

**FIGURE 1:**
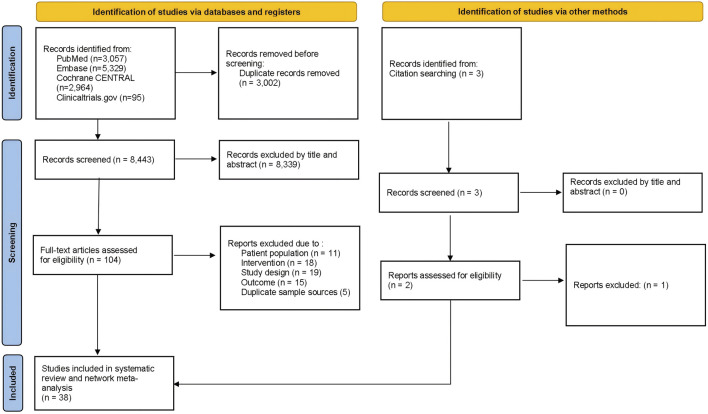
Flow Chart of the network meta-analysis procedure.

Figure 1 depicts the flowchart of the current study. Across the NMA, 11,445 database records were identified from the initial screening procedure for the review stage. Additional two pieces of literature were searched through citation searching. After removing duplicates, two independent authors were screened by title and abstract. Then, 104 articles were further screened by examining the full text, of which 67 were excluded for various reasons. Finally, 38 RCTs and 13,223 randomized participants (including the placebo group) were involved in the analysis. Supplementary Table S1 presents trials and the baseline demographic characteristics.

All included studies were published between 2004 and 2023 and conducted in several countries. We enrolled 12 classes of preventive medications, including memantine 10 mg (two trials, 109 participants), sodium valproate (one trials, 33 participants), topiramate 100 mg (six trials, 1,460 participants), propranolol 160 mg (two trials, 498 participants), amitriptyline 25 mg (one trial, 118 participants), candesartan 16 mg (two trials, 230 participants), rimegepant 75 mg (one trial, 741 participants), atogepant 10 mg (two trials, 722 participants), Erenumab 140 mg q4w (six trials, 2,505 participants), fremanezumab 225 mg q4w (six trials, 2,110 participants), galcanezumab 240 mg loading dose, 120 mg q4w (five trials, 2,707 participants), and Eptinezumab 300 mg, day 0 and week 12 (four trials, 1996 participants). Notably, some guideline-recommended drugs were not included in the current study. Because these drugs were tested decades ago, using partly different study designs, study populations and endpoints, they no longer meet the current NMA inclusion criteria. In addition, based on previous studies (Lampl et al., 2023), we did not include botulinum toxin in our analysis. Evidence suggests that the efficacy of botulinum toxin differs between chronic and episodic migraine (Herd et al., 2019; Herd et al., 2018), and FDA has only approved its use for the preventive treatment of chronic migraine. This study focuses on examining the relationship between memantine and other preventive treatments for migraine. Since the included memantine trials did not stratify patients by migraine type, we were unable to construct separate networks for chronic and episodic migraines. Including botulinum toxin under these circumstances would have introduced statistical heterogeneity. Other heterogeneity sources included techniques for injection and type of neurotoxin.

**FIGURE 2:**
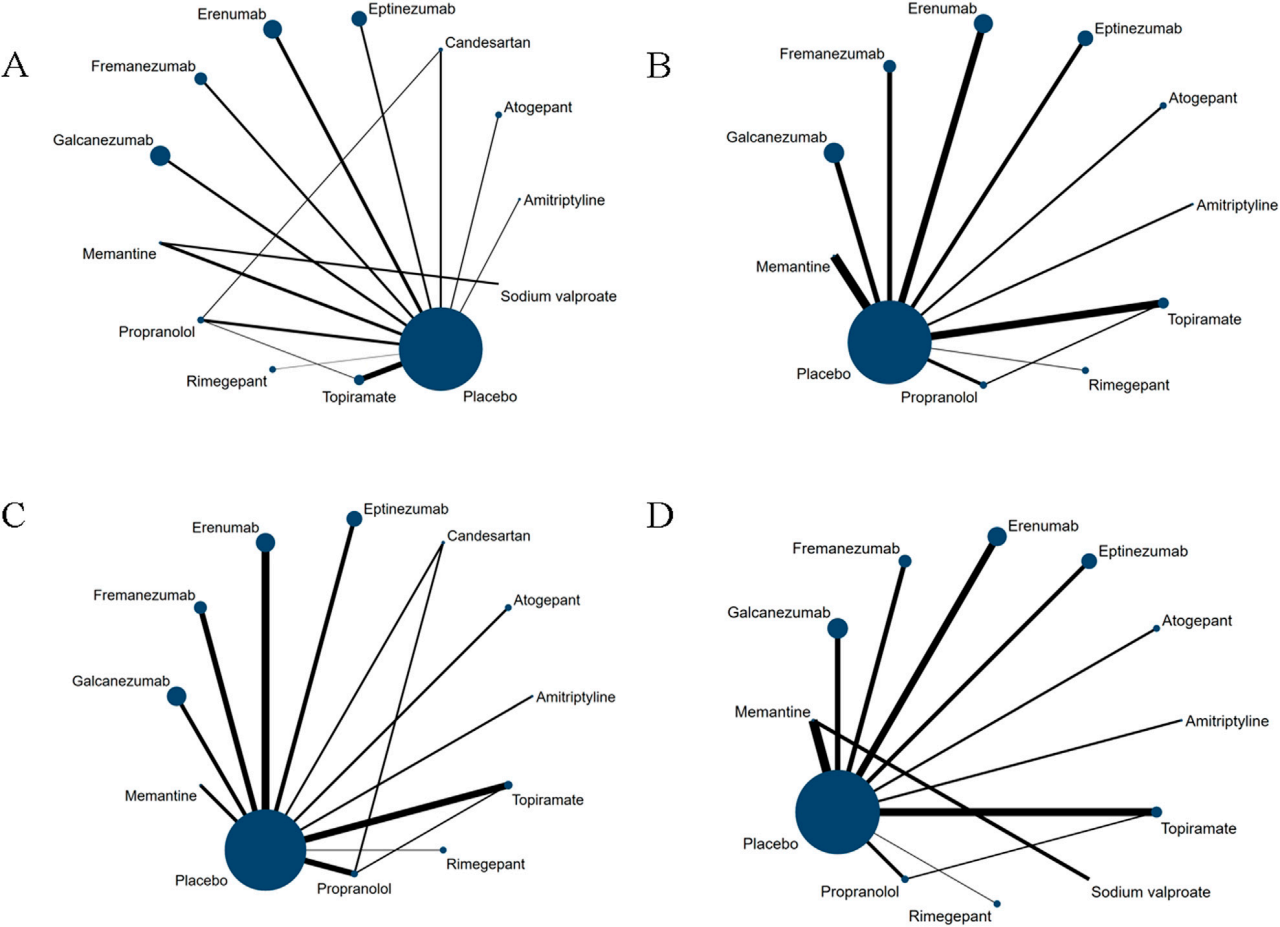
Network plot for primary outcomes and secondary outcomes. The lines between nodes represent direct comparisons in various trials, and each circle’s size is proportional to the population involved in each specific treatment. The thickness of the lines is proportional to the number of trials. **(A)** Changes in migraine days. **(B)** withdrawal due to adverse events. **(C)** 50% of the response rate. **(D)** Any adverse events within post-dose.

The overall geometric distribution of the treatment arms is provided in Figure 2. We labelled the heterogeneity statistic I^2^ of each outcome in the upper right of the forest plot and only detected substantial heterogeneity in the endpoint of response rate (I^2^ = 56.1%).

### Primary efficacy outcome: change from baseline in migraine days per month

**FIGURE 3:**
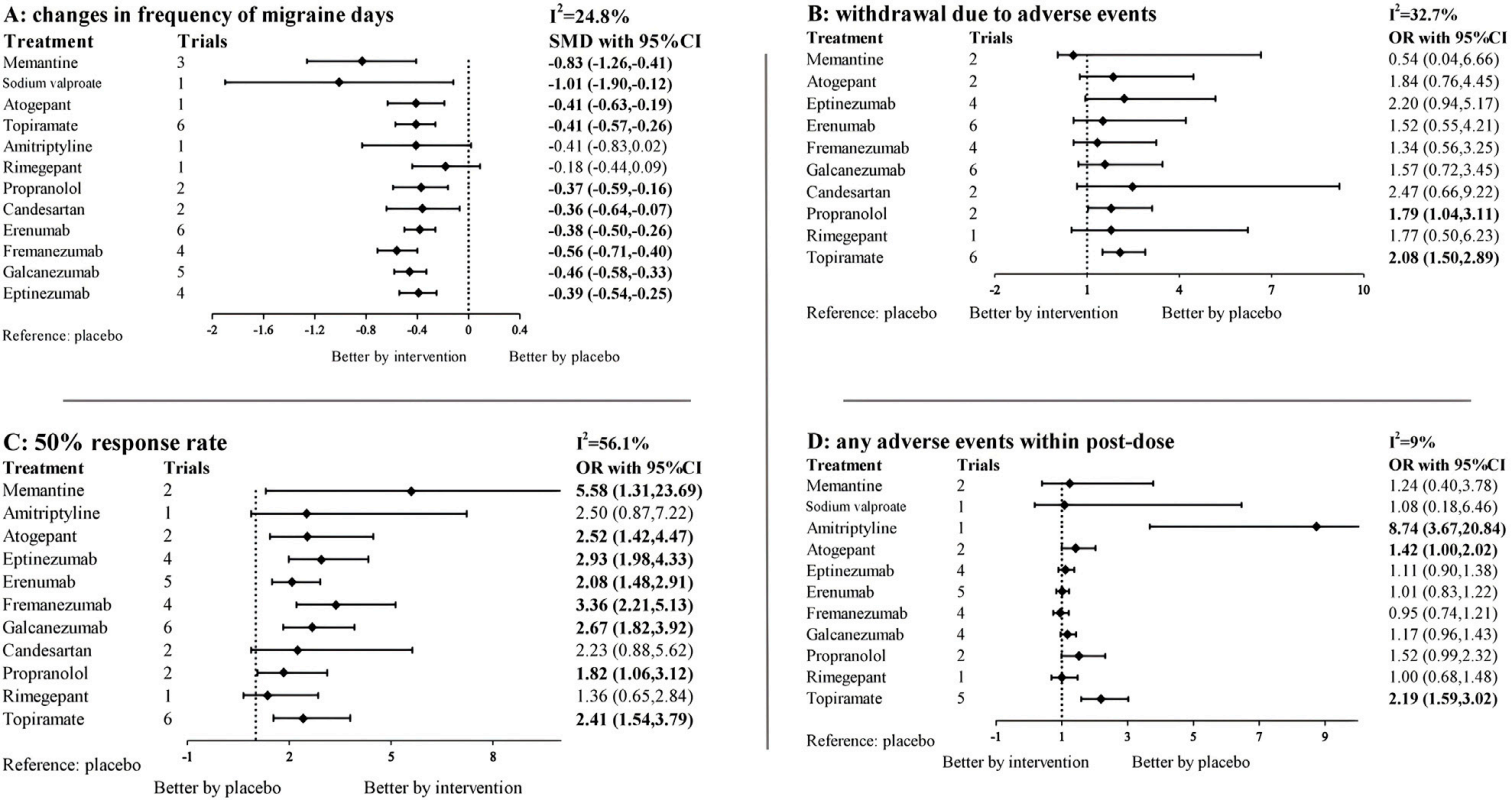
Forest plots for primary outcomes and secondary outcomes. SMD, standardized mean difference, OR, odds ratio, CI, confidence intervals. 95% CI. The forest plot was based on a random-effects model. Specific treatments were associated with **(A)** better improvement in migraine days than the placebo if the standardized mean difference was <0. For **(B–D)** 95% CI that did not contain one was considered statistically significant. For the effectiveness endpoint, results to the left of 1 favor placebo, to the right favor intervention, result in adverse events was the opposite.

The network diagram contains 38 studies and 12 individual pharmacological intervention nodes (Figure 2A). All remaining specific preventive drugs, except amitriptyline and rimegepant, were associated with significantly better improvements in migraine days than placebo, with sodium valproate showing the highest odds (SMD: −1.01; 95% CI: −1.90, −0.12), followed by memantine (SMD: −0.83; 95% CI: −1.26, −0.41), and rimegepant showing the lowest (SMD: −0.18; 95% CI: −0.44, 0.09) (Table 1; Figure 3A). In the base model, we found moderate and very low certainty evidence that memantine showed better improvements in migraine days than rimegepant (SMD: −0.66; 95% CI: −1.16, −0.16) and erenumab (SMD: −0.45; 95% CI: −0.90, −0.01). For most comparisons, the results were homogeneous in pairwise meta-analyses and network meta-analyses. According to the SUCRA, memantine was ranked as the best improvement in migraine days, followed by sodium valproate (Supplementary Table S2). The results of the subgroup and sensitivity analyses were broadly consistent with the trends of the base model (Supplementary Tables S11, S13).

**TABLE 1:** League table of changes in frequency of migraine days.

**Memantine**	0.17 (−0.61,0.96)											**−0.81 (−1.26,-0.42)**
0.18 (−0.61,0.96)	**Sodium valproate**											NA
−0.43 (−1.03,0.18)	−0.60 (−1.59,0.39)	**Amitriptyline**										−0.41 (-0.72,0.03)
−0.43 (−0.91,0.05)	−0.60 (−1.52,0.32)	−0.00 (−0.48,0.48)	**Atogepant**									**−0.39 (-0.50,-0.19)**
−0.48 (−0.99,0.04)	−0.65 (−1.59,0.29)	−0.05 (−0.56,0.46)	−0.05 (−0.41,0.31)	**Candesartan**							−0.06 (−0.18,0.34)	**−0.44 (-0.74,-0.13)**
−0.44 (−0.89,0.01)	−0.62 (−1.52,0.29)	−0.02 (−0.46,0.43)	−0.01 (−0.28,0.25)	0.03 (−0.29,0.36)	**Eptinezumab**							**−0.39 (-0.45,-0.21)**
−0.45 (−0.90,-0.01)	−0.63 (−1.53,0.27)	−0.03 (−0.47,0.41)	−0.03 (−0.28,0.22)	0.02 (−0.29,0.33)	−0.01 (−0.20,0.18)	**Erenumab**						**−0.35 (-0.49,-0.26)**
−0.28 (−0.73,0.17)	−0.45 (−1.36,0.45)	0.15 (−0.30,0.60)	0.15 (−0.12,0.42)	0.20 (−0.13,0.52)	0.16 (−0.05,0.37)	0.18 (−0.02,0.37)	**Fremanezumab**					**−0.56 (-0.71,-0.41)**
−0.38 (−0.82,0.07)	−0.55 (−1.46,0.35)	0.05 (−0.40,0.49)	0.05 (−0.21,0.30)	0.10 (−0.22,0.41)	0.06 (−0.13,0.26)	0.08 (−0.10,0.25)	−0.10 (−0.30,0.10)	**Galcanezumab**				**−0.44 (-0.55,-0.33)**
−0.46 (−0.94,0.02)	−0.64 (−1.56,0.28)	−0.03 (−0.51,0.44)	−0.03 (−0.34,0.28)	0.02 (−0.30,0.34)	−0.02 (−0.28,0.24)	−0.01 (−0.25,0.24)	−0.18 (−0.45,0.08)	−0.08 (−0.33,0.17)	**Propranolol**		−0.2 (−0.56,0.15)	−0.14 (−0.48,0.17)
−0.66 (−1.16,-0.16)	−0.83 (−1.77,0.10)	−0.23 (−0.73,0.27)	−0.23 (−0.57,0.11)	−0.18 (−0.57,0.21)	−0.21 (−0.52,0.09)	−0.20 (−0.49,0.09)	−0.38 (−0.68,-0.07)	−0.28 (−0.57,0.02)	−0.20 (−0.54,0.14)	**Rimegepant**		**−0.18 (-0.44,-0.09)**
−0.42 (−0.87,0.03)	−0.60 (−1.50,0.31)	0.01 (−0.45,0.46)	0.01 (−0.26,0.28)	0.06 (−0.26,0.38)	0.02 (−0.19,0.23)	0.03 (−0.16,0.23)	−0.14 (−0.36,0.07)	−0.04 (−0.24,0.16)	0.04 (−0.20,0.28)	0.24 (−0.07,0.54)	**Topiramate**	**−0.47 (-0.51,-0.22)**
**−0.83 (-1.26,-0.41)**	**−1.01 (-1.90,-0.12)**	−0.41 (−0.83,0.02)	**−0.41 (-0.63,-0.19)**	**−0.36 (-0.64,-0.07)**	**−0.39 (-0.54,-0.25)**	**−0.38 (-0.50,-0.26)**	**−0.56 (-0.71,-0.40)**	**−0.46 (-0.58,-0.33)**	**−0.37 (-0.59,-0.16)**	−0.18 (−0.44,0.09)	**−0.41 (-0.57,-0.26)**	**Placebo**

Pairwise (upper-right portion) and network (lower-left portion) meta-analysis results are presented as estimated effect size changes in the frequency of migraine days. For the result, outcomes are expressed as SMD, and 95% confidence interval. For the pairwise meta-analyses, SMD <0 indicates the treatment specified in the row showed better improvement in migraine attack frequency than that specified in the column. For the network meta-analysis, SMD <0 indicates the treatment specified in the column showed better improvement in migraine attack frequency than that specified in the row. Bold results indicated statistical significance.

### Primary safety outcome: withdrawal due to adverse events

We evaluated the withdrawal due to adverse events of the investigated pharmacologic interventions using the NMA. In brief, 33 studies and 10 treatments are included in this network (Figures 2B, 3B). Only topiramate (OR: 2.08; 95% CI: 1.50, 2.89) and propranolol (OR: 1.79; 95% CI: 1.04, 3.11) were associated with significantly higher dropout rates than the placebo (Table 2). The withdrawal due to adverse events for memantine appears low, but its confidence intervals contain null values. According to SUCRA (Supplementary Table S3), apart from the placebo, 10 mg of memantine was associated with the lowest dropout rate, followed by Fremanezumab, and candesartan ranked the highest. In the subgroup and sensitivity analyses (Supplementary Tables S12, S14), the primary outcome remained consistent with the basic model, with only topiramate and propranolol showing a higher dropout rate than placebo.

**TABLE 2:** League table of withdrawal due to adverse events.

**Memantine**										0.54 (0.04,6.62)
0.29 (0.02,4.20)	**Atogepant**									1.84 (0.77,4.35)
0.24 (0.02,3.47)	0.83 (0.25,2.84)	**Eptinezumab**								2.18 (0.95,5.01)
0.35 (0.02,5.34)	1.21 (0.31,4.67)	1.45 (0.38,5.49)	**Erenumab**							1.53 (0.55,4.20)
0.40 (0.03,5.75)	1.37 (0.39,4.76)	1.64 (0.48,5.56)	1.13 (0.29,4.35)	**Fremanezumab**						1.34 (0.56,3.19)
0.34 (0.02,4.76)	1.17 (0.36,3.80)	1.40 (0.44,4.43)	0.97 (0.27,3.50)	0.85 (0.26,2.77)	**Galcanezumab**					1.57 (0.72,3.39)
0.22 (0.01,3.73)	0.75 (0.15,3.63)	0.89 (0.19,4.26)	0.62 (0.12,3.26)	0.54 (0.11,2.65)	0.64 (0.14,2.95)	**Candesartan**	1.08 (0.21,5.56)			3.22 (0.64,12.32)
0.30 (0.02,3.94)	1.03 (0.36,2.89)	1.23 (0.45,3.34)	0.85 (0.27,2.71)	0.75 (0.27,2.11)	0.88 (0.34,2.27)	1.38 (0.37,5.12)	**Propranolol**		0.71 (0.41,1.23)	**2.24(1.22,4.10)**
0.30 (0.02,5.07)	1.04 (0.22,4.84)	1.25 (0.27,5.70)	0.86 (0.17,4.34)	0.76 (0.16,3.54)	0.89 (0.20,3.92)	1.40 (0.23,8.63)	1.01 (0.26,4.01)	**Rimegepant**		1.77 (0.51,6.10)
0.26 (0.02,3.27)	0.88 (0.34,2.27)	1.06 (0.42,2.66)	0.73 (0.25,2.13)	0.65 (0.25,1.66)	0.76 (0.32,1.78)	1.19 (0.31,4.52)	0.86 (0.50,1.49)	0.85 (0.23,3.13)	**Topiramate**	**2.06(1.51,2.82)**
0.54 (0.04,6.66)	1.84 (0.76,4.45)	2.20 (0.94,5.17)	1.52 (0.55,4.21)	1.34 (0.56,3.25)	1.57 (0.72,3.45)	2.47 (0.66,9.22)	**1.79 (1.04,3.11)**	1.77 (0.50,6.23)	**2.08 (1.50,2.89)**	**Placebo**

Pairwise (upper-right portion) and network (lower-left portion) meta-analysis results are presented as estimated effect sizes for withdrawal due to adverse events. For the result, outcomes are expressed as odds ratios (OR) with a 95% confidence intervals (CI). For the pairwise meta-analyses, OR < 1 indicates the treatment specified in the row had a lower dropout rate than that specified in the column. For the network meta-analysis, OR < 1 indicates the treatment specified in the column had a lower dropout rate than that specified in the row. 95% CI, that did not contain one was considered to have a statistical difference. Bold results indicated statistical significance.

### Secondary efficacy outcome: response rate of a 50% reduction in monthly migraine days

The network diagram and forest plot are shown in Figures 2C, 3C. The detailed NMA results are shown in Supplementary Table S10. In brief, the network diagram shows 35 trials and 11 intervention nodes. All specific preventive drugs, except amitriptyline, candesartan and rimegepant, were associated with significantly higher 50% response rates than placebo. Memantine appears to have the highest ORs versus placebo, but the confidence intervals are extensive (OR: 5.58; 95% CI: 1.31, 23.69). According to the SUCRA, memantine was associated with the highest response rate of all the pharmacologic interventions, followed by fremanezumab and eptinezumab (Supplementary Table S4).

### Secondary safety outcome: frequency of any adverse event

Amitriptyline (OR: 8.74; 95% CI: 3.67, 20.84), atogepant (OR: 1.42; 95% CI: 1.00, 2.02), and topiramate (OR: 2.19; 95% CI: 1.59, 3.02) were associated with a significantly higher frequency of any adverse event during the pharmacologic intervention than placebo (Figures 2D, 3D). Direct and mixed comparisons were generally consistent except for Propranolol. According to the SUCRA, except for placebo, Fremanezumab was associated with the lowest frequency of any adverse event of all the investigated treatment arms, followed by Erenumab and rimegepant (Supplementary Table S5).

### Publication bias and inconsistency

In the risk of bias assessment, 83.7% (31/38 items) and 16.3% (7/38 items) had an overall low and some concerns about the risk of bias. No studies were assessed to be at high risk of bias (Supplementary Tables S1, S2). The measurement of the outcome and selection of the reported result mainly contributed to some concerns. The funnel plot of publication bias across the included studies showed moderate levels of symmetry (Supplementary Table S3), and the results of the Egger tests did not detect any significant publication bias (all *p* > 0.05). There was no significant global inconsistency in the design-by-treatment model (Supplementary Tables S6). The local inconsistency test revealed nonsignificant inconsistency in the present NMA (Supplementary Tables S7, S8). The GRADE results assessment is presented in Appendix S3. Overall, the quality of evidence for most comparisons in the current NMA ranged from low to very low.

## Discussion

Memantine is an antagonist of the NMDA subtype of the glutamate receptor. Glutamate levels are significantly elevated in the plasma and cerebrospinal fluid of patients with migraine during attacks (Ferrari et al., 1990; Martinez et al., 1993). Monosodium glutamate consumption can trigger headache attacks (Baad-Hansen et al., 2010). Glutamate has been discussed as a critical neurotransmitter in the central sensitisation mechanism of migraine (Hoffmann and Charles, 2018). Glutamate receptors, including all three classes of ionotropic glutamate receptors (iGluR) and metabotropic glutamate receptors (mGluR), are expressed in the trigeminocervical complex (TCC) (Tallaksen-Greene et al., 1992). The TCC serves as a major relay center that integrates peripheral and central components of the trigeminal vascular pathway, playing a pivotal role in migraine development. The microiontophoretic administration of glutamate into the Sp5C of the TCC can facilitate neuronal facilitation in this region (Hoffmann and Charles, 2018). These emphasise the potential importance of glutamate in the central transmission of injurious trigeminal signals. Additionally, glutamate is strongly implicated in cortical spreading depression (CSD), the primary event believed to underlie migraine aura (Podkowa et al., 2023). Excessive glutamate release disrupts brainstem activity—particularly in the periaqueductal grey, nucleus raphe magnus, and locus coeruleus—altering cortical function and increasing susceptibility to CSD (Podkowa et al., 2023; Fejes et al., 2011; Costa et al., 2013). In a rodent model, memantine can inhibit CSD. Therefore, the use of memantine to target excessive glutamatergic signalling as a therapeutic strategy for migraine makes sense.

Despite memantine is a promising prophylactic medication for migraine, it has only been tested in few studies. Noruzzadeh et al. (2016) conducted the first randomized, double-blind, placebo-controlled trial to assess its efficacy in migraine prevention (Noruzzadeh et al., 2016). A total of 52 migraine patients were included in the final analysis. Patients in the memantine group were titrated from 5 to 10 mg per night after 3 days. After 12 weeks, memantine showed superior efficacy compared to placebo in reducing migraine frequency (1.9 ± 0.4 vs 3.7 ± 0.4) and migraine days (2.5 ± 0.9 vs 8.4 ± 0.9). Shanmugam et al. (Shanmugam et al., 2019) extended the treatment period to 24 weeks, with 60 patients randomly assigned to receive either 10 mg of memantine daily or a matching placebo. Both groups experienced a decline in migraine days during the first 12 weeks (memantine: 10.79 days–5.18 days; placebo: 10.14 days–6.03 days). However, only the memantine group continued to improve beyond Week 12, with migraine frequency decreasing fourfold by Week 24 (from 10.79 days at baseline to 2.57 days), whereas no further decline was observed in the placebo group between 12 and 24 weeks. No serious adverse events were reported in either group throughout the study. Only two pilot studies have directly compared memantine and sodium valproate in migraine prevention. Damaris et al. (Vazquez-Guevara et al., 2023) conducted a 12-week prospective, randomized, double-blind, controlled clinical trial in Mexico involving 33 migraine patients, with 27 completing the study. After 12 weeks, the mean monthly migraine frequency decreased by 4.21 (SD ± 1.76) in the memantine group and 4.5 (SD ± 1.39) in the sodium valproate group. No serious adverse events were reported in either group, with somnolence being the most common side effect in both groups. In another cross-over clinical trial involving 70 patients with chronic or episodic migraine (Ghorbani et al., 2015), no significant difference in migraine frequency was observed between the memantine and sodium valproate groups after 12 weeks of treatment. However, the memantine group showed a significantly greater reduction in headache intensity compared to the sodium valproate group.

Although the prophylactic effect of memantine has been preliminarily demonstrated, these studies are limited by small sample sizes and lack of long-term efficacy and safety data, undermining confidence in its use for migraine management. The results of this NMA indicate that memantine presents non-inferior preventive effects compared with both traditional preventive medications and the new CGRP-mAbs in terms of efficacy and acceptability. Specifically, memantine showed the second-largest reduction in migraine days, the highest 50% response rate, the fewest adverse events leading to discontinuation, and a moderate frequency of adverse events compared to placebo. Commonly used agents, such as propranolol and topiramate, appear not only to be less effective than memantine but they are associated with a significantly increased risk of adverse events. However, it is noteworthy that many confidence intervals include null values, which may be related to the small number of studies included and insufficient sample sizes, particularly in three studies on memantine. This limitation reduces the statistical power and may lead to imprecise estimates of effect sizes, affecting the reliability of the NMA findings.

An additional advantage of memantine is its excellent safety and tolerability profile. Memantine is recognised as a pregnancy category B drug and has been used for decades for the approved indication of Alzheimer’s disease. In this NMA, despite memantine showed a moderate frequency of adverse events, few participants terminated the trial due to side effects. The most commonly reported adverse effects—such as somnolence, sedation, and nausea—were mild (Bigal et al., 2008). Regarding current literature, memantine appears to be a safe and effective option for adult migraine. However, the safety data is limited to adverse events leading to treatment discontinuation and common side effects for short-term use. To date, no studies have conducted long-term follow-up to evaluate its preventive effects. Possible tolerance, dependence, or rare adverse reactions—such as severe allergic responses or autoimmune diseases—remain unclear. Additionally, the interactions between memantine and patients’ genetic characteristics, chronic conditions, or concomitant medications are not well understood. Future prospective cohort studies with longer follow-up durations are necessary to address these gaps.

Overall, memantine effectively reduces the frequency of migraine attacks, improves response rates, and demonstrates good safety and tolerability. It may be particularly suitable for migraine patients with cognitive impairments. Memantine is also a viable option in migraine patients for whom conventional preventive medications are ineffective, intolerable, contraindicated, or unavailable for novel migraine medications. Some studies have shown that memantine has potential benefits for conditions (Strzelecki et al., 2013; Kishi et al., 2018; Serra et al., 2014) such as depression, bipolar disorder, and schizophrenia. For migraine patients with comorbid psychiatric conditions, memantine may not only prevent migraines but also provide auxiliary benefits in emotional regulation. Additionally, memantine has a distinct mechanism of action compared to conventional migraine preventive drugs. Therefore, its combination with other preventive therapies, including CGRP-mAbs and gepants, is also worth considering as a possible therapy for refractory migraine patients. However, despite the potential benefits of memantine in migraine prevention, researchers and companies still appear to lack sufficient interest in investigating memantine as a migraine therapy. A reason is that memantine has established clear indications, which may cause pharmaceutical companies to lack sufficient incentives to bear the risk and expense of conducting clinical trials for a different indication like migraine. The development of novel CGRP-mAbs has further decreased motivation to explore alternative treatments like memantine. Nonetheless, the current pilot NMA supports the prioritisation of memantine in migraine prevention medications. We hope this study encourages further research into the potential role of memantine in migraine management and provides a rationale for conducting future large-scale randomized controlled trials (RCTs) to thoroughly investigate its effectiveness.

### Strengths and limitations

This research has several strengths. Firstly, the NMA allows us to estimate the relative potency of all the interventions considered and establish a ranking order for them by dissecting direct and indirect evidence from different studies, even in the absence of head-to-head trials. Second, we included only RCTs and trials using placebo. Given the higher placebo effect (Faria et al., 2014) in pain and headache studies (40%–55%), this initiative improves the reliability of the current findings. Third, no RCTs included in the analysis were assessed at high risk of bias. The results of the NMA depend significantly on the quality and heterogeneity of the included studies. We used strict inclusion criteria. Although it led to certain commonly used drugs were not included in the analyses, this improves the quality of the evidence for the NMA.

Our results should be approached with caution due to several limitations. First, although we used strict inclusion and exclusion criteria to improve the homogeneity of the included studies, some potential heterogeneity between studies with respect to participant characteristics will inevitably remain (e.g., baseline age differences, the proportion of headache severity, combined medications, underlying diseases and the presence or absence of migraine aura). Secondly, the small sample sizes of the three studies on memantine may result in insufficient statistical power and unstable estimates of effect sizes, potentially exaggerating the relative efficacy of memantine, particularly when employing random effects models. Third, the three studies of memantine were conducted in India, Mexico and Iran. Only one prospective open-label study was conducted in the USA, meaning that the results may not be replicable to a broader population. This may be due to the stringent regulations in Western countries concerning off-label prescriptions. Patients can only consider memantine after attempting other first-line medications. Additionally, in recent years, new migraine-specific medications, like CGRP-mAbs and gepants, have rapidly gained traction in Europe and the United States, which has undoubtedly reduced the adoption of memantine in migraine treatment. For most developing countries, the high cost of novel migraine drugs still largely limits their widespread use, prompting a greater willingness to explore off-label experiences with memantine for migraine treatment. However, the pharmacokinetic properties and drug response of memantine may be influenced by factors such as drug metabolism-related genes, comorbidities, diet, stress levels, and climate in different populations. Therefore, it is essential to include a broader and more diverse sample representing various ethnic and cultural backgrounds in future studies. Fourth, efficacy and safety outcomes of memantine have been evaluated between 4 and 24 weeks, while data beyond 6 months have not been established. In addition, few head-to-head studies of memantine were included, thus most comparisons were derived from indirect estimates, which may reduce the precision of the estimates. Fifth, by means of cumulative probabilities, SUCRA can visualise the probability distribution of different treatments across various ranking positions, facilitating consistent interpretation of NMA (Rosenberger et al., 2021). In the current NMA, the SUCRA rankings suggest that memantine may be the best option for reducing migraine days. However, The reliance on SUCRA rankings to declare memantine as the best treatment might be overstated given the small sample sizes and the lack of indirect comparisons.

## Conclusion

This pilot NMA demonstrated that memantine was associated with a significant reduction in migraine frequency, improved response rates, and fair acceptability. These beneficial effects were not inferior to current pharmacologic regimens approved by the FDA or treatment guidelines. Memantine may be particularly suitable for migraine patients with cognitive impairments. It is also a viable therapy option for patients in whom previous treatments are ineffective, intolerable, contraindicated, or unavailable for novel migraine medications. However, limited by the relatively small number of studies and sample sizes, many confidence intervals contain null values. Furthermore, given the absence of long-term efficacy, safety data and direct comparisons with active control agents, the estimates of memantine may be overly optimistic. Clinicians should avoid over-interpreting the findings of current NMAs and apply them in a relatively conservative manner. Future research should include larger samples, multicente, longer durations and using active controls to further assess the long-term efficacy and safety of memantine.

## Data Availability

The original contributions presented in the study are included in the article/Supplementary Material, further inquiries can be directed to the corresponding authors.

## Abbreviations

NMA, Network meta-analysis; NMDA, N-methyl-d-aspartate; CINeMA, Confidence in Network Meta-Analysis; FDA, Food and Drug Administration; ICHD, International Classification of Headache Disorders criteria; SUCRA, Surface under the cumulative ranking curve; DALYs, Disability-adjusted life years; YLD, Years lived with disability; CGRP-mAbs, Calcitonin gene-related peptide monoclonal antibodies; CGRP, Calcitonin gene-related peptide; IHS, International Headache Society; GRADE, Grading of Recommendations Assessment, Development, and Evaluation, TCC, Trigeminocervical complex; CSD, Cortical spreading depression.
