# Supplementary material for: Role of memantine in adult migraine: a systematic review and network meta-analysis to compare memantine with existing migraine preventive medications
Source: Front Pharmacol. 2024 Dec 18;15:1496621. doi: 10.3389/fphar.2024.1496621 (PMC11688350; doi:10.3389/fphar.2024.1496621)
Supplement: Supplementary file 1 [file DataSheet1.docx]

# **Supplementary material**

Appendix 1. PRISMA checklist of the current network meta-analysis

Appendix 2. Search strategies

Appendix 3. GRADE ratings for primary outcome network

Table S1. Baseline demographics characteristics

Table S2-5. SUCRA of each outcome

Table S6. Design-by-treatment interaction model for the inconsistency of network meta-analysis

Table S7. Significant loop-specific inconsistencies of network meta-analysis

Table S8. Significant side-splitting inconsistencies of network meta-analysis

Table S9. League table of 50% response rate

Table S10. League table of frequency of any adverse event

Table S11-12. Subgroup analysis for primary efficacy and safety

Table S13-14. Sensitivity analyses for primary efficacy and safety

Figure S1. Overview of risk of bias

Figure S2. Detailed risk of bias in each study

Figure S3. Funnel plot of changes in each outcome

References

This supplementary material has been provided by the authors to give readers additional information about their work.

**Appendix 1:** PRISMA checklist of the current network meta-analysis

| **Section and Topic** | **Item #** | **Checklist item** | **Location where item is reported** |
| --- | --- | --- | --- |
| **TITLE** | | |  |
| Title | 1 | Identify the report as a systematic review incorporating a network meta-analysis (or related form of meta-analysis) | 1 |
| **ABSTRACT** | | |  |
| Abstract | 2 | See the PRISMA 2020 for Abstracts checklist. | 1 |
| **INTRODUCTION** | | |  |
| Rationale | 3 | Describe the rationale for the review in the context of existing knowledge. | 2-3 |
| Objectives | 4 | Provide an explicit statement of the objective(s) or question(s) the review addresses. | 2-3 |
| **METHODS** | | |  |
| Eligibility criteria | 5 | Specify the inclusion and exclusion criteria for the review and how studies were grouped for the syntheses. | 3-4 |
| Information sources | 6 | Specify all databases, registers, websites, organisations, reference lists and other sources searched or consulted to identify studies. Specify the date when each source was last searched or consulted. | 3 |
| Search strategy | 7 | Present the full search strategies for all databases, registers and websites, including any filters and limits used. | 3-4 |
| Selection process | 8 | Specify the methods used to decide whether a study met the inclusion criteria of the review, including how many reviewers screened each record and each report retrieved, whether they worked independently, and if applicable, details of automation tools used in the process. | 4-5 |
| Data collection process | 9 | Specify the methods used to collect data from reports, including how many reviewers collected data from each report, whether they worked independently, any processes for obtaining or confirming data from study investigators, and if applicable, details of automation tools used in the process. | 4-5 |
| Data items | 10a | List and define all outcomes for which data were sought. Specify whether all results that were compatible with each outcome domain in each study were sought (e.g. for all measures, time points, analyses), and if not, the methods used to decide which results to collect. | 4-5 |
|  | 10b | List and define all other variables for which data were sought (e.g. participant and intervention characteristics, funding sources). Describe any assumptions made about any missing or unclear information. | 5 |
| Study risk of bias assessment | 11 | Specify the methods used to assess risk of bias in the included studies, including details of the tool(s) used, how many reviewers assessed each study and whether they worked independently, and if applicable, details of automation tools used in the process. | 5 |
| Effect measures | 12 | Specify for each outcome the effect measure(s) (e.g. risk ratio, mean difference) used in the synthesis or presentation of results. | 5-6 |
| Synthesis methods | 13a | Describe the processes used to decide which studies were eligible for each synthesis (e.g. tabulating the study intervention characteristics and comparing against the planned groups for each synthesis (item #5)). | 5-6 |
|  | 13b | Describe any methods required to prepare the data for presentation or synthesis, such as handling of missing summary statistics, or data conversions. | 5-6 |
|  | 13c | Describe any methods used to tabulate or visually display results of individual studies and syntheses. | 5-6 |
|  | 13d | Describe any methods used to synthesize results and provide a rationale for the choice(s). If meta-analysis was performed, describe the model(s), method(s) to identify the presence and extent of statistical heterogeneity, and software package(s) used. | 5-6 |
|  | 13e | Describe any methods used to explore possible causes of heterogeneity among study results (e.g. subgroup analysis, meta-regression). | 5-6 |
|  | 13f | Describe any sensitivity analyses conducted to assess robustness of the synthesized results. | 5-6 |
| Reporting bias assessment | 14 | Describe any methods used to assess risk of bias due to missing results in a synthesis (arising from reporting biases). | 6 |
| Certainty assessment | 15 | Describe any methods used to assess certainty (or confidence) in the body of evidence for an outcome. | 6 |
| **RESULTS** | | |  |
| Study selection | 16 | Describe the results of the search and selection process, from the number of records identified in the search to the number of studies included in the review, ideally using a flow diagram. | 6-8  Figure 1  Appendix2 |
| Study characteristics | 17 | Cite each included study and present its characteristics. | 6-7  Table S1 |
| Risk of bias in studies | 18 | Present assessments of risk of bias for each included study. | Figure S1, 2 |
| Results of individual studies | 19 | For all outcomes, present, for each study: (a) summary statistics for each group (where appropriate) and (b) an effect estimate and its precision (e.g. confidence/credible interval), ideally using structured tables or plots. | Figure 3 |
| Results of syntheses | 20a | For each synthesis, briefly summarise the characteristics and risk of bias among contributing studies. | 7-10  Figure S1, 2 |
|  | 20b | Present results of all statistical syntheses conducted. If meta-analysis was done, present for each the summary estimate and its precision (e.g. confidence/credible interval) and measures of statistical heterogeneity. If comparing groups, describe the direction of the effect. | 7-10  Table 1 Table S9-10 |
|  | 20c | Present results of all investigations of possible causes of heterogeneity among study results. | Figure 3 |
|  | 20d | Present results of all sensitivity analyses conducted to assess the robustness of the synthesized results. | Table 11-12 |
| Reporting biases | 21 | Present assessments of risk of bias due to missing results (arising from reporting biases) for each synthesis assessed. | Figure S1-2 |
| Certainty of evidence | 22 | Present assessments of certainty (or confidence) in the body of evidence for each outcome assessed. | Appendix 3 |
| **DISCUSSION** | | |  |
| Discussion | 23a | Provide a general interpretation of the results in the context of other evidence. | 10-13 |
|  | 23b | Discuss any limitations of the evidence included in the review. | 14 |
|  | 23c | Discuss any limitations of the review processes used. | 14 |
|  | 23d | Discuss implications of the results for practice, policy, and future research. | 14 |
| **OTHER INFORMATION** | | |  |
| Registration and protocol | 24a | Provide registration information for the review, including register name and registration number, or state that the review was not registered. | 3 |
|  | 24b | Indicate where the review protocol can be accessed, or state that a protocol was not prepared. | not prepared |
|  | 24c | Describe and explain any amendments to information provided at registration or in the protocol. | not prepared |
| Support | 25 | Describe sources of financial or non-financial support for the review, and the role of the funders or sponsors in the review. | 16 |
| Competing interests | 26 | Declare any competing interests of review authors. | 16 |
| Availability of data, code and other materials | 27 | Report which of the following are publicly available and where they can be found: template data collection forms; data extracted from included studies; data used for all analyses; analytic code; any other materials used in the review. | 16 |

**Appendix 2:** Search strategies

**PubMed from inception until 1 June 2024**

| # | Searches | Results |
| --- | --- | --- |
| 1 | Migraine Disorders[Mesh] | 33,130 |
| 2 | Migraine disorders[Title/Abstract] OR Disorder, Migraine[Title/Abstract] OR Disorders, Migraine[Title/Abstract] OR Migraine Disorder[Title/Abstract] OR Migraine[Title/Abstract] OR Migraines[Title/Abstract] OR Migraine Headache[Title/Abstract] OR Headache, Migraine[Title/Abstract] OR Headaches, Migraine[Title/Abstract] OR Migraine Headaches[Title/Abstract] OR Acute Confusional Migraine[Title/Abstract] OR Acute Confusional Migraines[Title/Abstract] OR Migraine, Acute Confusional[Title/Abstract] OR Migraines, Acute Confusional[Title/Abstract] OR Status Migrainosus[Title/Abstract] OR Hemicrania Migraine[Title/Abstract] OR Hemicrania Migraines[Title/Abstract] OR Migraine, Hemicrania[Title/Abstract] OR Migraines, Hemicrania[Title/Abstract] OR Migraine Variant[Title/Abstract] OR Migraine Variants[Title/Abstract] OR Variant, Migraine[Title/Abstract] OR Variants, Migraine[Title/Abstract] OR Sick Headache[Title/Abstract] OR Headache, Sick[Title/Abstract] OR Headaches, Sick[Title/Abstract] OR Sick Headaches[Title/Abstract] OR Abdominal Migraine[Title/Abstract] OR Abdominal Migraines[Title/Abstract] OR Migraine, Abdominal[Title/Abstract] OR Migraines, Abdominal[Title/Abstract] OR Cervical Migraine Syndrome[Title/Abstract] OR Cervical Migraine Syndromes[Title/Abstract] OR Migraine Syndrome, Cervical[Title/Abstract] OR Migraine Syndromes, Cervical[Title/Abstract] | 45,299 |
| 3 | 1 OR 2 | 49,518 |
| 4 | topiramate OR topamax OR valproate OR valproic acid OR propranolol OR propanolol OR Metoprolol OR timolol OR amitriptyline OR venlafaxine OR flunarizine OR frovatriptan OR candesartan | 106,286 |
| 5 | cgrp monoclonal antibodies OR calcitonin gene-related peptide antibodies (cgrp) monoclonal antibodies (mabs) OR cgrp mabs OR rimegepant OR Atogepant OR Erenumab OR Fremanezumab OR Galcanezumab OR Eptinezumab | 1,418 |
| 6 | 4 OR 5 | 107,640 |
| 7 | (clinical[tiab] AND trial[tiab]) OR clinical trials as topic[mesh] OR clinical trial[pt] OR random*[tiab] OR random allocation[mesh] OR therapeutic use[sh] | 6,579,235 |
| 8 | animals[Mesh] NOT humans[Mesh] | 5,245,597 |
| 9 | 7 NOT 8 | 5,779,185 |
| 10 | 3 AND 6 AND 9 | 3,057 |

**Embase from inception until 1 June 2024**

| **#** | **Searches** | **Results** |
| --- | --- | --- |
| 1 | 'migraine disorders'/exp | 84,865 |
| 2 | 'migraine disorders':ab,ti,kw OR 'disorder, migraine':ab,ti,kw OR 'disorders, migraine':ab,ti,kw OR 'migraine disorder':ab,ti,kw OR 'migraine':ab,ti,kw OR 'migraines':ab,ti,kw OR 'migraine headache':ab,ti,kw OR 'headache, migraine':ab,ti,kw OR 'headaches, migraine':ab,ti,kw OR 'migraine headaches':ab,ti,kw OR 'acute confusional migraine':ab,ti,kw OR 'acute confusional migraines':ab,ti,kw OR 'migraine, acute confusional':ab,ti,kw OR 'migraines, acute confusional':ab,ti,kw OR 'status migrainosus':ab,ti,kw OR 'hemicrania migraine':ab,ti,kw OR 'hemicrania migraines':ab,ti,kw OR 'migraine, hemicrania':ab,ti,kw OR 'migraines, hemicrania':ab,ti,kw OR 'migraine variant':ab,ti,kw OR 'migraine variants':ab,ti,kw OR 'variant, migraine':ab,ti,kw OR 'variants, migraine':ab,ti,kw OR 'sick headache':ab,ti,kw OR 'headache, sick':ab,ti,kw OR 'headaches, sick':ab,ti,kw OR 'sick headaches':ab,ti,kw OR 'abdominal migraine':ab,ti,kw OR 'abdominal migraines':ab,ti,kw OR 'migraine, abdominal':ab,ti,kw OR 'migraines, abdominal':ab,ti,kw OR 'cervical migraine syndrome':ab,ti,kw OR 'cervical migraine syndromes':ab,ti,kw OR 'migraine syndrome, cervical':ab,ti,kw OR 'migraine syndromes, cervical':ab,ti,kw | 71,436 |
| 3 | 1 OR 2 | 93,456 |
| 4 | 'topiramate':ab,ti,kw OR 'topamax':ab,ti,kw OR 'valproate':ab,ti,kw OR 'valproic acid':ab,ti,kw OR 'propranolol':ab,ti,kw OR 'propanolol':ab,ti,kw OR 'Metoprolol':ab,ti,kw OR 'timolol':ab,ti,kw OR 'intranasal drug administrations':ab,ti,kw OR 'amitriptyline':ab,ti,kw OR 'venlafaxine':ab,ti,kw OR 'flunarizine':ab,ti,kw OR 'candesartan':ab,ti,kw | 121,844 |
| 5 | 'cgrp monoclonal antibodies':ab,ti,kw OR 'calcitonin gene-related peptide antibodies (cgrp) monoclonal antibodies (mabs)':ab,ti,kw OR 'cgrp mabs':ab,ti,kw OR 'rimegepant':ab,ti,kw OR 'Atogepant':ab,ti,kw OR 'Erenumab':ab,ti,kw OR 'Fremanezumab':ab,ti,kw OR 'Galcanezumab':ab,ti,kw OR 'Eptinezumab':ab,ti,kw | 3,446 |
| 6 | 4 OR 5 | 125,112 |
| 7 | 'clinical':ti,ab AND 'trial':ti,ab OR 'clinical trial'/exp OR random* OR 'drug therapy':lnk | 7,377,771 |
| 8 | 3 AND 6 AND 7 | 5,329 |

**Cochrane Library from inception until 1 June 2024**

| **#** | **Searches** | **Results** |
| --- | --- | --- |
| 1 | MeSH descriptor: [Migraine disorders] explode all trees | 3,904 |
| 2 | migraine disorders OR disorder, migraine OR disorders, migraine OR migraine disorder OR migraine OR migraines OR migraine headache OR headache, migraine OR headaches, migraine OR migraine headaches OR acute confusional migraine OR acute confusional migraines OR migraine, acute confusional OR migraines, acute confusional OR status migrainosus OR hemicrania migraine OR hemicrania migraines OR migraine, hemicrania OR migraines, hemicrania OR migraine variant OR migraine variants OR variant, migraine OR variants, migraine OR sick headache OR headache, sick OR headaches, sick OR sick headaches OR abdominal migraine OR abdominal migraines OR migraine, abdominal OR migraines, abdominal OR cervical migraine syndrome OR cervical migraine syndromes OR migraine syndrome, cervical OR migraine syndromes, cervical | 10,592 |
| 3 | 1 OR 2 | 10,592 |
| 4 | topiramate OR topamax OR valproate OR valproic acid OR propranolol OR propanolol OR Metoprolol OR timolol OR amitriptyline OR venlafaxine OR flunarizine OR frovatriptan OR candesartan | 21,806 |
| 5 | cgrp monoclonal antibodies OR calcitonin gene-related peptide antibodies (cgrp) monoclonal antibodies (mabs) OR cgrp mabs OR rimegepant OR Atogepant OR Erenumab OR Fremanezumab OR Galcanezumab OR Eptinezumab | 2,335 |
| 6 | 4 OR 5 | 24,063 |
| 8 | 3 AND 6 | 3,067 |
| 9 | In trials | 2,964 |

**Appendix 3:** GRADE ratings for primary outcome network

The Grading of Recommendations Assessment, Development, and Evaluation (GRADE) methods provide confidence assessments of the results of systematic reviews and meta-analyses and have been widely accepted and applied [1, 2]. However, the complexity of the methods and the lack of suitable software have limited their adoption. Confidence in Network Meta-Analysis (CINeMA) [3] is an approach for determining confidence in the results of an NMA broadly based on GRADE, with several conceptual and semantic differences. It covers 6 domains: (A) within-study bias, (B) reporting bias, (C) indirectness, (D) imprecision, (E) heterogeneity, and (F) incoherence. Only the reviewer needs to input the within-study bias and indirectness at the study level. The three levels (no concerns, some concerns, or major concerns) for each domain can be judged according to user-defined rules. The judgments for each domain are eventually summarized to obtain 4 levels of confidence for each pair of comparisons (very low, low, moderate, or high). we examined the certainty of the evidence for the network estimate in line with the following criteria:

Within-study bias: CINeMA combines the studies’ contributions with the risk of bias judgments to evaluate within-study bias for each estimate from an NMA. It uses the percentage contribution matrix to approximate the contribution of each study. Then it computes the percentage contribution from studies judged to be at low, moderate, and high risk of bias. We categorized the overall risk of bias in each study. Then based on the results of the Cochrane Risk of Bias version 2 (RoB2) for randomized trials [4], we allocate the values of “low,” “some concern,” and “high,” as 1, 2, and 3, respectively, and input into the CINeMA web application [5].

Reporting bias: Although the CINeMA approach suggested some conditions associated with suspected reporting bias, this is still highly subjective. Therefore, by referring to previous NMA studies [6, 7], a comparison-adjusted funnel plot with an accompanying Egger test for asymmetry was calculated.

Indirectness: We judged that there was no concern in this domain as the included studies matched our inclusion criteria and study questions.

Imprecision: CINeMA compares the treatment effects included in the 95% confidence interval with the range of equivalence. Due to the absence of previous analyses that could be referenced, we considered a clinically meaningful threshold for odds ratios (OR) to be 0.

Heterogeneity: As for imprecision, the CINeMA approach to heterogeneity involves comparisons of results with the pre-specified range of clinical equivalence.

Incoherence: As for heterogeneity, the CINeMA approach to incoherence considers the impact on clinical implications based on visual inspection of the 95% confidence intervals of direct and indirect ORs and the range of equivalence.

Summarizing judgments across the 6 domains: For each pair comparison, one may start at high confidence and drop the confidence level by 1 step for each domain with some concerns, and by 2 levels for each domain with major concerns. However, it is essential to note that domains are interconnected. The 6 CINeMA domains should therefore be considered jointly rather than in isolation, avoiding downgrading the overall level of confidence more than once for related concerns. Therefore, in the GRADE of the current NMA, for the first three domains, the downgrading was no more than two levels; the same was performed for the last three domains.

**Primary efficacy outcome: change from baseline in migraine days per month**


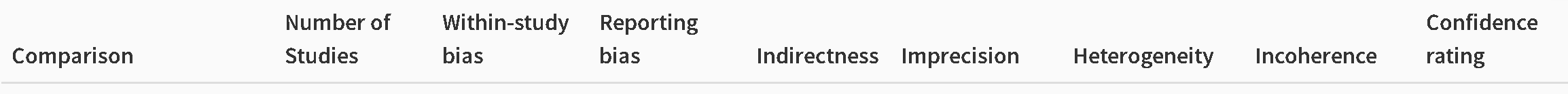


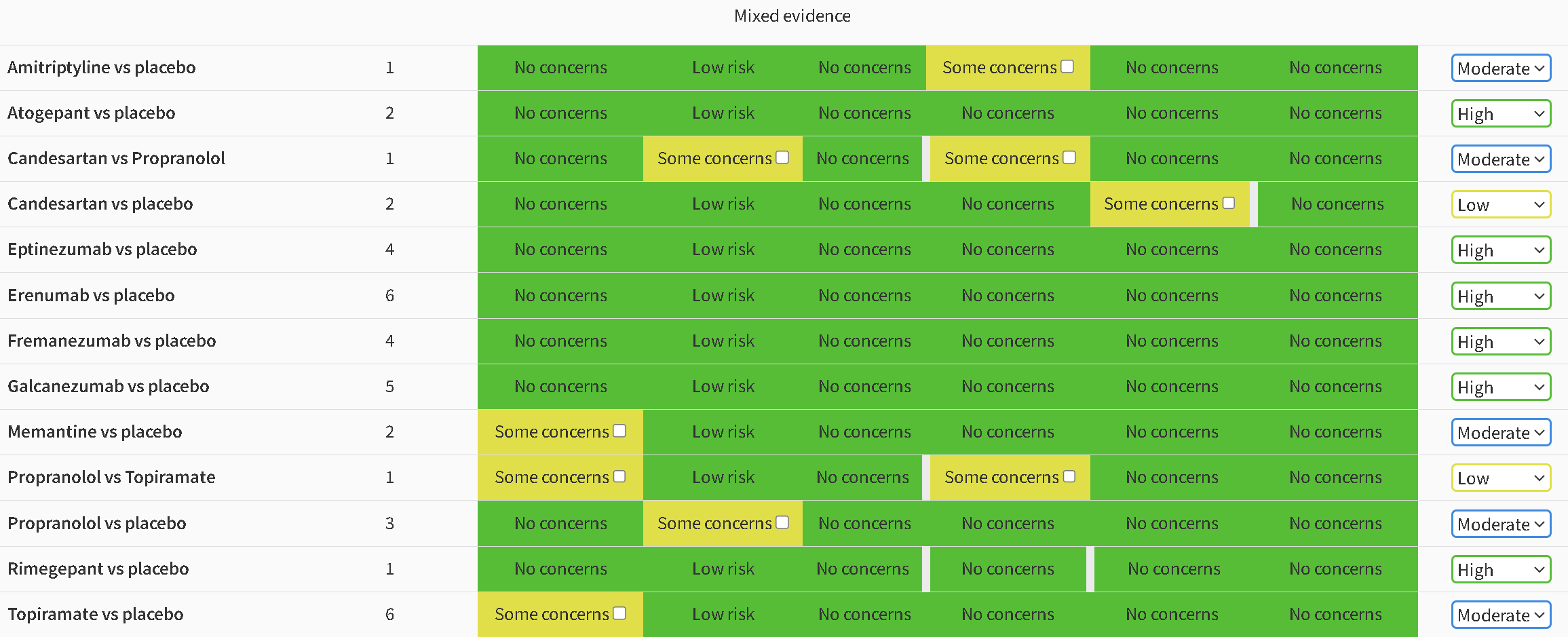


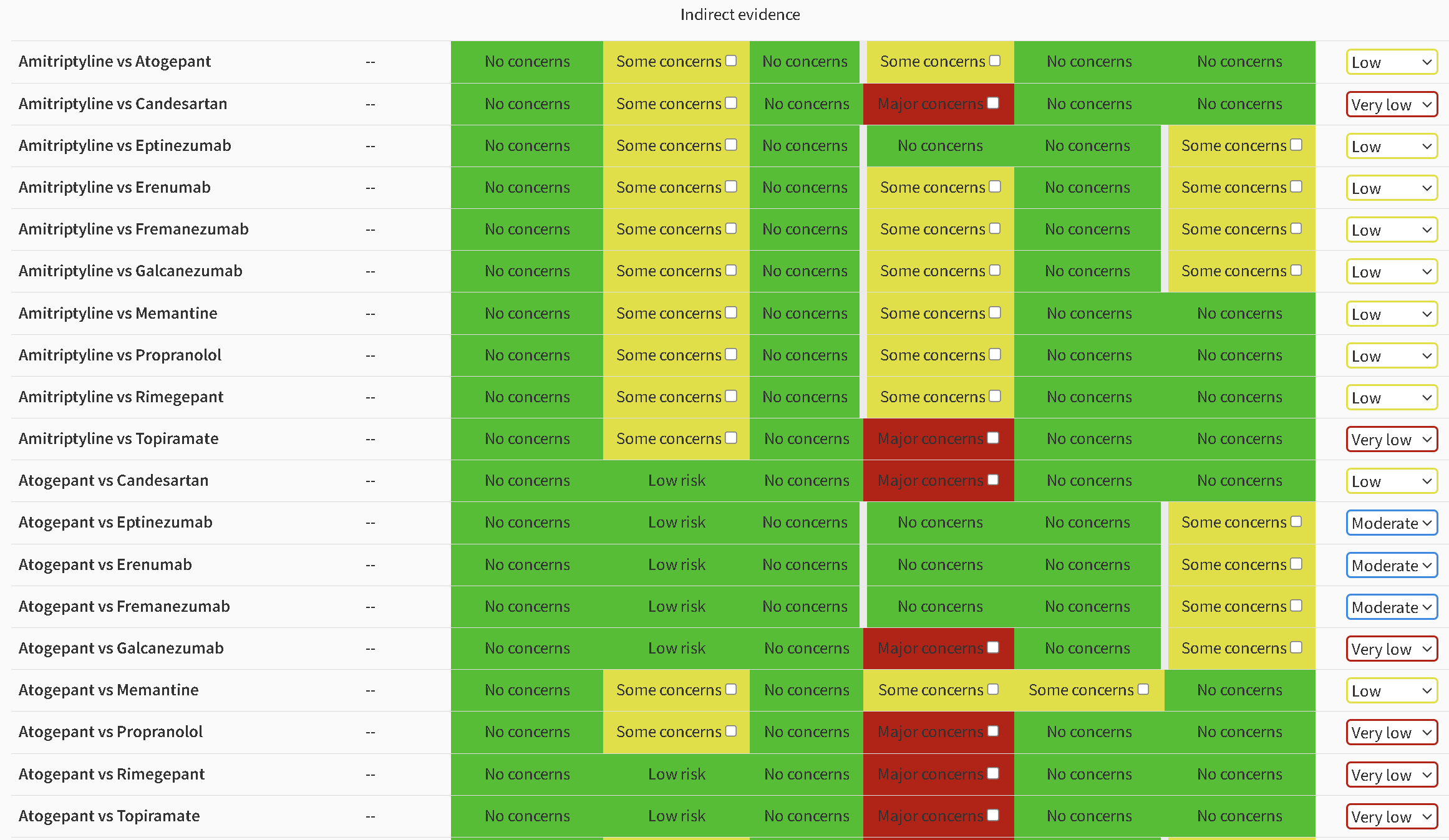


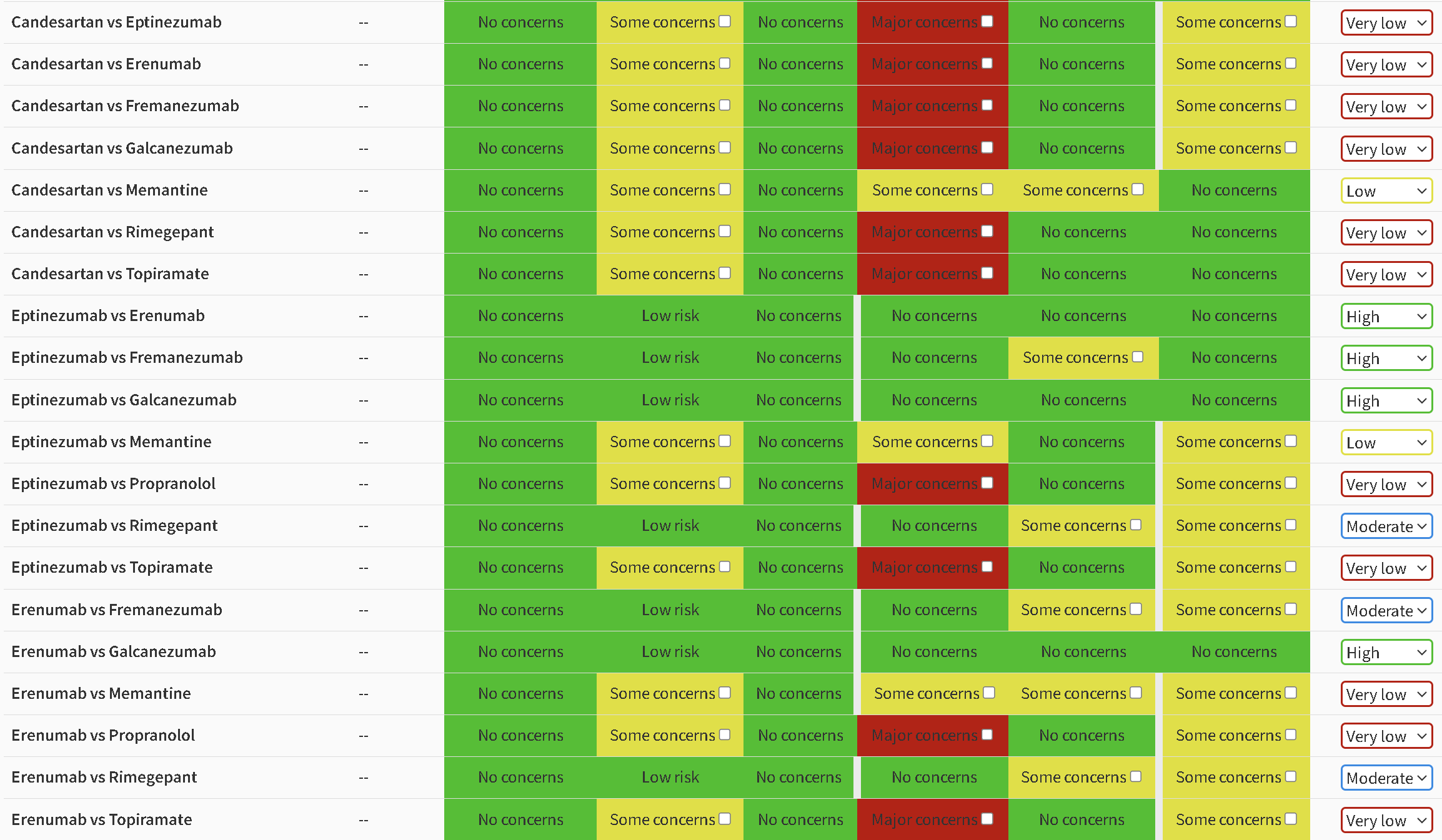


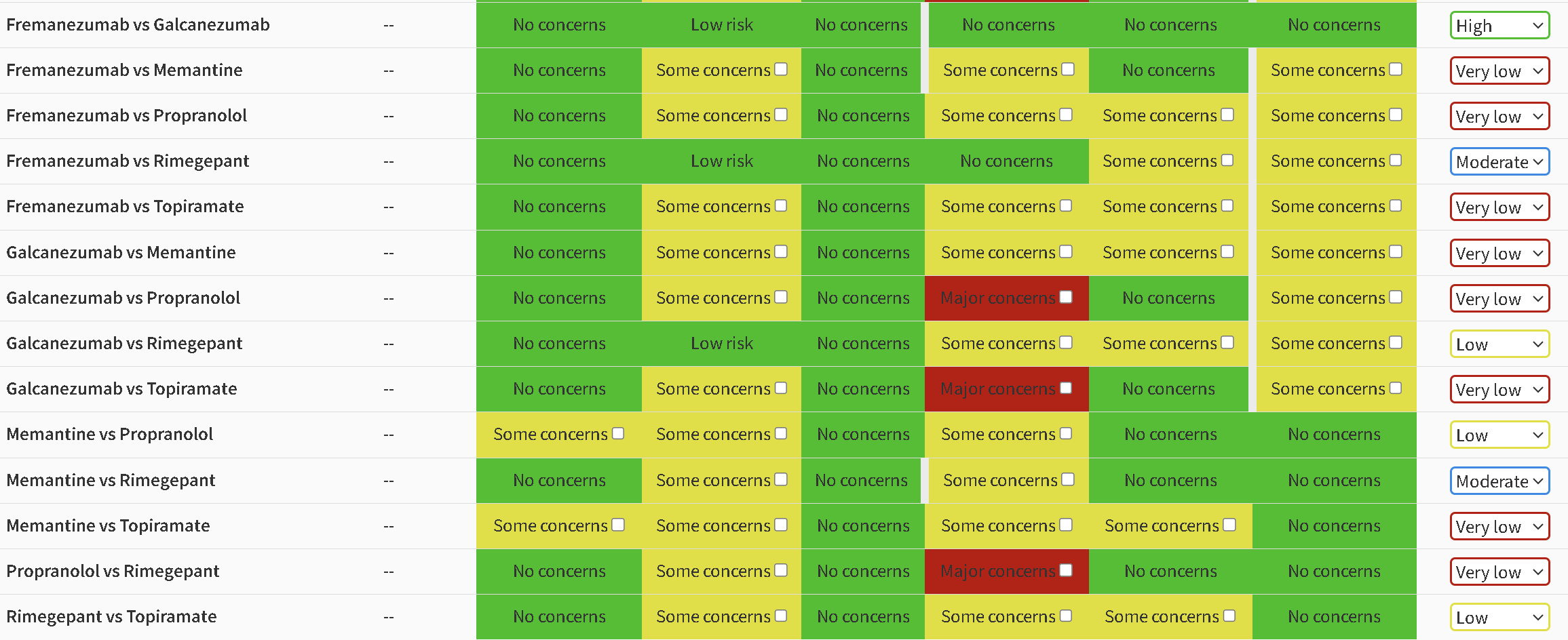


**Primary safety outcome: withdrawal due to adverse events**


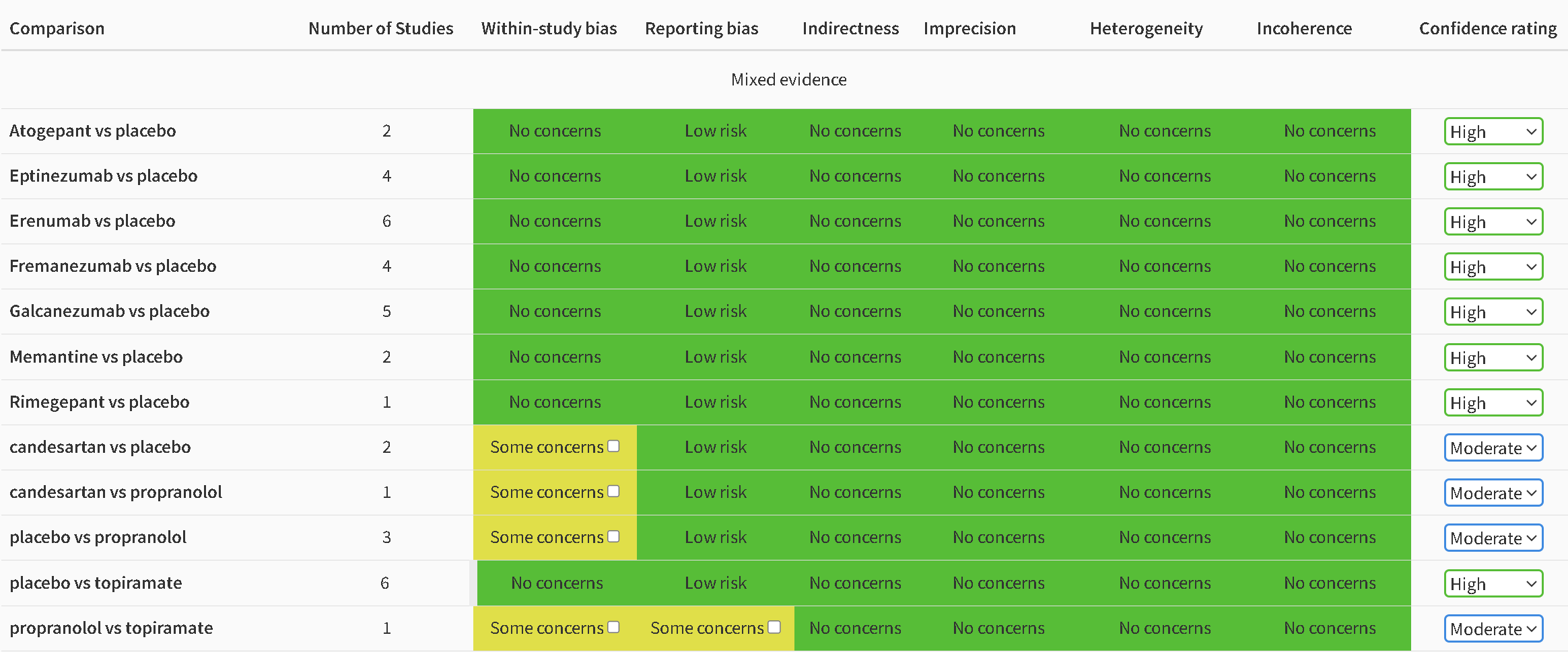


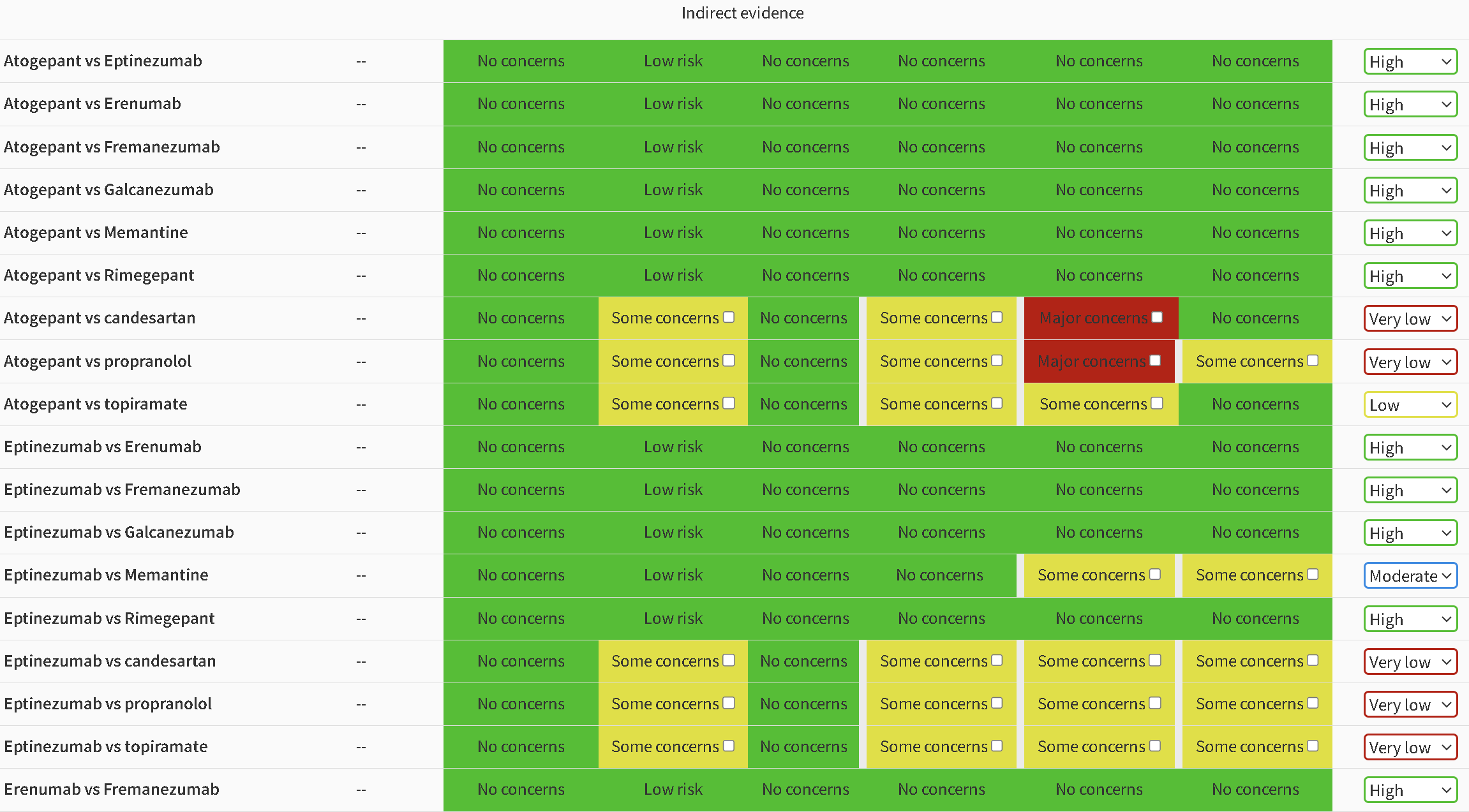


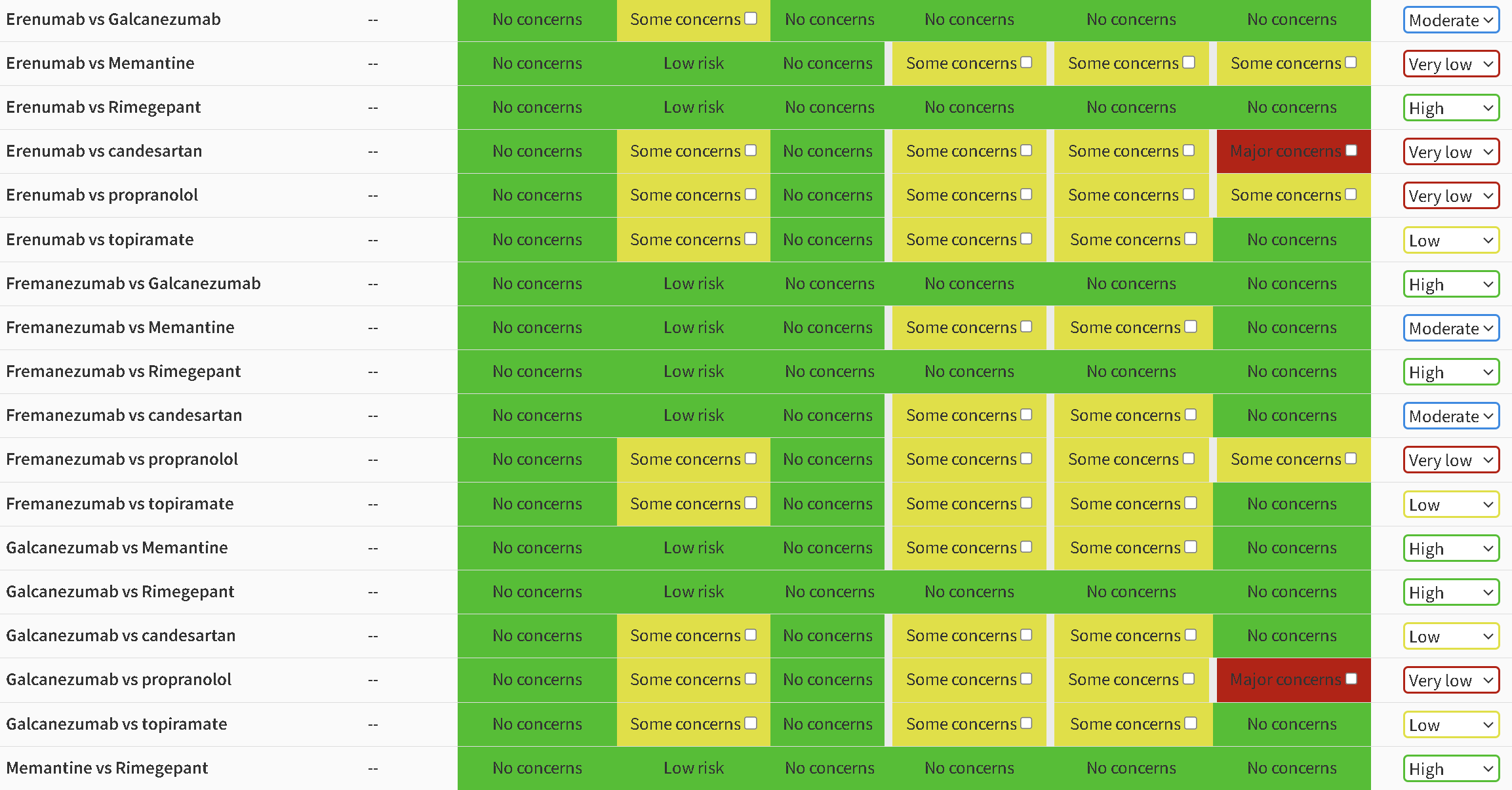


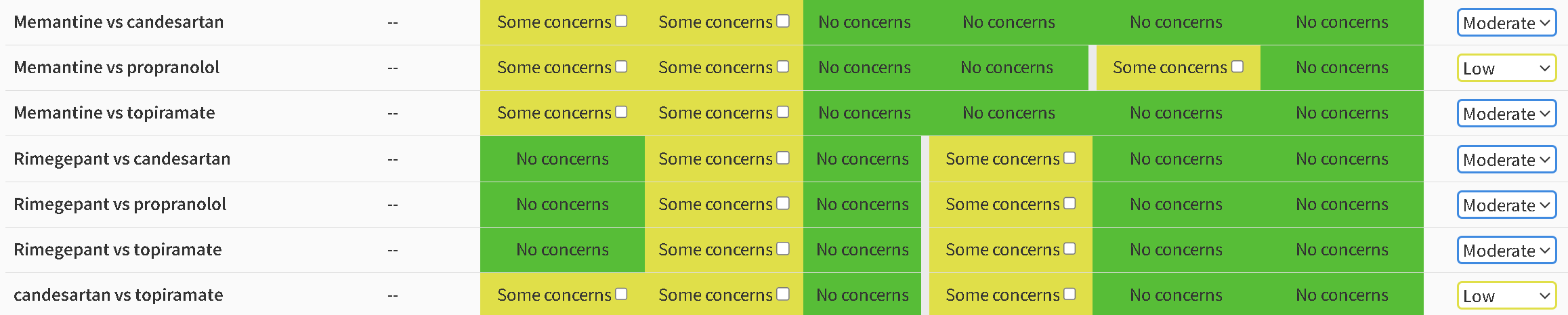


**Table S1.** Baseline demographics characteristics

| **Studies included** | **Registration** | **Country,** | **Diagnostic criteria** | **Intervention, Regimens, and routes** | **Sample** | **female, %** | **Mean age (years, mean ±SD)** | **Duration of Migraine (years)** | **Migraine/ Headache Days Per Month(mean ±SD)** | **Risk of bias** |
| --- | --- | --- | --- | --- | --- | --- | --- | --- | --- | --- |
|  |  | **Study Design,** |  |  |  |  |  |  |  |  |
|  |  | **Study Period** |  |  |  |  |  |  |  |  |
| Noruzzadeh2016 [8] | IRCT2013120115616N1 | RCT in the Iran, 02/2014 to 09/2014 | ICHD-2 | Memantine 10mg for 12 weeks, oral/ placebo | 52 | 77% | 34.8 ± 7.4 | NR | 5.4 ± 2.5 | Low |
| Shanmugam, S2019 [9] | CTRI/2013/11/004172 | RCT in India | IHS | Memantine 10mg for 12 weeks, oral/ placebo | 57 | 66% | 18-30 | NR | NR | Some concerns |
| Goadsby, P. J2020 [10] | NCT02848326 | RCT in the USA, 09/2016 to 04/2018 | ICHD-3 | Atogepant 30 mg for 12 weeks, oral/ placebo | 369 | 85% | 41.0 ± 13.6 | 19.9± (13.7) | NR | Low |
| Ailani, J et al.2021 [11] | NCT03777059 | RCT in the USA, 12/2018 to 06/2020 | ICHD-3 | Atogepant 30 mg for 12 weeks, oral/ placebo | 460 | 89% | 42.1±11.7 | NR | 7.3±2.4 | Low |
| Silberstein, S2009 [12] | CR004684 | RCT in the USA | IHS | topiramate 100mg for 16 weeks, oral/ placebo | 182 | NR | 38.2±12.1 | NR | NR | Some concerns |
| Diener, H. C2007 [13] | NR | RCT in the Germany, 11/2003 to 07/2005 | IHS | topiramate 100mg for 16 weeks, oral/ placebo | 59 | 75% | 47.8 ± 9.4 | NR | 15.5 ± 4.6 | Some concerns |
| Lipton, R. B2011 [14] | CR002854 | RCT in multiple countries, 9/2005 to 08/2007 | ICHD-2 | topiramate 100mg for 26 weeks, oral/ placebo | 385 | 87% | 39.6 ±10.6 | 19.8± 10 | 13.0± 2.5 | Some concerns |
| Diener, H. C.2004 [15] | NR | RCT in multiple countries | IHS | topiramate 100mg for 16 weeks, oral/ propranolol 160mg for 16 weeks, oral/placebo | 575 | 85% | 34.6 ±5.7 | NR | 12.5 ± 3.9 | Some concerns |
| Silberstein, S. D.2004 [16] | NR | RCT in the USA | IHS | topiramate 100mg for 36 weeks, oral/ placebo | 242 | 92% | 40.6 ±11.1 | NR | 6.4±2.7 | Low |
| Brandes, J. L.2004 [17] | NR | RCT in North America | IHS | topiramate 100mg for 26 weeks, oral/ placebo | 126 | 71% | 32.6 ±12.5 | NR | 11.1±5.3 | Some concerns |
| Goncalves, A. L2016 [18] | NCT01357031 | RCT in multiple countries | ICHD-3 | amitriptyline 25 mg for 12 weeks, oral/ placebo | 118 | 75% | 37.2±11.2 | 24.1±9.1 | 7.2±2.5 | Low |
| Croop, R.2021 [19] | NCT03732638 | RCT in the USA, 11/2018 to 08/2019 | ICHD-3 | rimegepant 75 mg for 12 weeks, oral/ placebo | 695 | 81% | 41.3±13 | NR | 10.1±3.1 | Low |
| Holroyd, K. A.2010 [20] | NCT00910689 | RCT in the USA, 07/2001 to 11/2005 | IHS | propranolol for 68 weeks, oral/ placebo | 108 | 76% | NR | NR | 8.5±6.7 | Low |
| Tronvik, E.2003 [21] | NR | RCT in the Norway, 01/2001 to 02/2002 | IHS | candesartan 16mg for 12weeks/ placebo | 57 | 82% | NR | NR | 13.6±10.7 | Low |
| Stovner, L. J.2014 [22] | NCT00884663 | RCT in the Norway, 04/2009 to 03/2012 | IHS | candesartan 16 mg, propranolol slow-release 160 mg, or placebo for 12weeks | 72 | 84% | 37±11 | 19±11 | 4.8±3.6 | Some concerns |
| Tepper, S.2017 [23] | NCT02066415 | RCT in multiple countries, 04/2014 to 12/2015 | ICHD-3 | Erenumab 70mg q4w for 12 weeks, SC/ placebo | 477 | 87% | 41.4±11.3 | 20.7±12.8 | 17.9±4.4 | Low |
| Sun, H.2016 [24] | NCT01952574 | RCT in multiple countries, 08/2013 to 06/2014 | ICHD-3 | Erenumab 70mg q4w for 12 weeks, SC/ placebo | 267 | 77% | 42.6±9.9 | 21.5±11.7 | 8.9±2.9 | Low |
| Wang, S. J.2021 [25] | NCT03333109 | RCT in multiple countries, 02/2018 to 01/2020 | ICHD-3 | Erenumab 70mg q4w for 12 weeks, SC/ placebo | 776 | 80% | 37.3±10 | 11.1±9.5 | 8.1±2.6 | Low |
| Takeshima.2021 [26] | NCT03812224 | RCT in Japan, 04/2019 to 02/2020 | ICHD-3 | Erenumab 70 mg q4w for 24 weeks, SC/placebo | 261 | 85% | 44.2±8.5 | NR | 12.1±5.4 | Low |
| Dodick, D. W.2018 [27] | NCT02483585 | RCT in multiple countries, 07/2015 to 07/2016 | ICHD-3 | Erenumab 70mg q4w for 12 weeks, SC/ placebo | 517 | 86% | 42±11 | 22±13 | 8.1±2.7 | Low |
| Sakai, F.2019 [28] | NCT02630459 | RCT in Japan | ICHD-3 | Erenumab 70mg q4w for 12 weeks, SC/ placebo | 271 | 85% | 44 | NR | 7.8±2.3 | Low |
| Bigal, M. E.2015 [29] | NCT02021773 | RCT in the USA | ICHD-2 | Fremanezumab 225mg q4w for 12 weeks, SC/ placebo | 200 | 91% | 40.8±12.4 | 18.9±12.9 | 11.5±1.9 | Low |
| Ferrari, M. D.2019 [30] | NCT03308968 | RCT in multiple countries, 11/2017 to 07/2018 | ICHD-3 | Fremanezumab 225mg q4w for 12 weeks, SC/ placebo | 562 | 84% | 45.9±11.1 | NR | 14.1±5.6 | Low |
| Silberstein, S. D.2017 [31] | NCT02621931 | RCT in multiple countries, 03/2016 to 01/2017 | ICHD-3 | Fremanezumab 675mg q4w for 12 weeks, SC/ placebo | 754 | 88% | 40.6±12 | NR | 16.4±5.2 | Low |
| Sakai, F.2021 [32] | NCT03303092 | RCT in Japan and Korea, 1/2017 to 11/2019 | ICHD-3 | Fremanezumab 225mg q4w for 12 weeks, SC/ placebo | 381 | 84% | 44.4±9.5 | 22.0±12.9 | 8.6±2.5 | Low |
| Sakai, F.2021 [33] | NCT03303079 | RCT in Japan and Korea, 11/2017 to 11/2019 | ICHD-3 | Fremanezumab 675mg q4w for 12 weeks, SC/ placebo | 380 | 86% | 42.7±10.2 | 18.3±12.4 | 16.4±5.3 | Low |
| Dodick, D. W.2018 [34] | NCT02629861 | RCT in multiple countries, 03/2016 to 04/2017 | ICHD-3 | Fremanezumab 225mg q4w for 12 weeks, SC/ placebo | 584 | 84% | 42.9±12.7 | NR | 8.9±2.6 | Low |
| Dodick, D. W.2014 [35] | NCT01625988 | RCT in the USA, 07/2012 to 09/2013 | ICHD-3 | Galcanezumab 150 mg, q2w for 12 weeks, SC/placebo | 329 | 79% | 38.8±12.3 | NR | 7.3±3.8 | Low |
| Mulleners, W. M.2020 [36] | NCT03559257 | RCT in multiple countries, 09/2018 to 03/2019 | ICHD-3 | Galcanezumab 240 mg loading dose, 120 mg q4w for 12 weeks, SC/Placebo | 462 | 84% | 41.6±13.5 | 14.0±12.1 | 9.1±2.4 | Low |
| Hu, B.2020 [37] | NCT03963232 | RCT in China, India and Russia, 07/2012 to 09/2013 | ICHD-3 | Galcanezumab 240 mg loading dose, 120 mg q4w for 12 weeks, SC/Placebo | 512 | 73% | 37.2±9.3 | 12.8±9.2 | 8.2±2.8 | Low |
| Detke, H. C.2018 [38] | NCT02614261 | RCT in multiple countries | ICHD-3 | Galcanezumab 120 mg for 12 weeks, SC/Placebo | 835 | 84% | 39.7±10.2 | 10.3±8.4 | 11.4±5.9 | Low |
| Skljarevski, V.2018 [39] | NCT02614196 | RCT in multiple countries | ICHD-3 | Galcanezumab 120 mg for 12 weeks, SC/Placebo | 684 | 71% | 42.7±10.1 | NR | 7.4±6.5 | Low |
| Skljarevski, V.2018 [40] | NCT02163993 | RCT in the USA | ICHD-3 | Galcanezumab 120 mg for 12 weeks, SC/Placebo | 215 | 81% | 39.2±9.3 | 10.8±9.1 | 6.2±3.5 | Low |
| Ashina, M.2022 [41] | NCT04418765 | RCT in multiple countries | ICHD-3 | Eptinezumab 300mg, day 0 and week 12, IV/Placebo | 594 | 79% | 38.9±11.8 | NR | 7.6±1.5 | Low |
| Ashina, M.2020 [42] | NCT02559895 | RCT in the USA | ICHD-3 | Eptinezumab 300 mg, q12w, IV/Placebo | 508 | 83% | 35.2±7.4 | 11.5±6.4 | 6.8±5.5 | Low |
| Dodick, D. W.2019 [43] | NCT02275117 | RCT in multiple countries | ICHD-3 | Eptinezumab 300 mg, q12w, IV/Placebo | 208 | 77% | 40.6±9.1 | 9.0±5.4 | 4.2±3.8 | Low |
| Lipton, R. B.2020 [44] | NCT02974153 | RCT in multiple countries, 11/2016 to 04/2018 | ICHD-3 | Eptinezumab 300mg, day 0 and week 12, IV/Placebo | 716 | 89% | 41.0±10.4 | 19.0±11.5 | 16.1±4.8 | Low |
| Damaris, 2023 [45] | NCT04698525 | RCT in Mexico, 7/2019 to 10/2020 | ICHD-3 | 10 mg memantine tablet per night for one week, followed by an increase to one 10 mg tablet morning and night; 250 mg  valproate tablet per night for one week, followed by an increase to one 250 mg tablet morning and evening with a maximum dose of 500 mg | 87 | 78% | 31.1±10.9 | 10 | 5.31±1.54 | Low |

| IHS, international headache society; ICHD, International Classification of Headache Disorders; ICHD-2, International Classification of Headache Disorders, 2nd edition; ICHD-3, International Classification of Headache Disorders, 3rd edition. |  |
| --- | --- |

**Table S2:** SUCRA of change from baseline in migraine days per month

| **Treatment** | **SUCRA, n%** |
| --- | --- |
| Memantine | 96.1 |
| Sodium valproate | 94.2 |
| Fremanezumab | 83.0 |
| Galcanezumab | 64.7 |
| Topiramate | 53.6 |
| Amitriptyline | 52.3 |
| Atogepant | 52.1 |
| Eptinezumab | 47.9 |
| Propranolol | 45.1 |
| Erenumab | 44.9 |
| Candesartan | 42.8 |
| Rimegepant | 6.4 |
| Placebo | 1.2 |

SUCRA values vary from 0 to 1, whereas 1 means the treatment might be the best and 0 the worst.

**Table S3:** SUCRA of adverse events leading to discontinuation

| **Treatment** | **SUCRA, n%** |
| --- | --- |
| Placebo | 16.9 |
| Memantine | 19.9 |
| Fremanezumab | 39.4 |
| Erenumab | 46.7 |
| Galcanezumab | 48.1 |
| Rimegepant | 55.3 |
| Propranolol | 56.4 |
| Atogepant | 58.3 |
| Topiramate | 68.5 |
| Eptinezumab | 69.5 |
| Candesartan | 71.0 |

SUCRA values vary from 0 to 1, whereas 1 means the highest dropout rate.

**Table S4:** SUCRA of 50% response rate

| **Treatment** | **SUCRA, n%** |
| --- | --- |
| Memantine | 86.7 |
| Fremanezumab | 79.4 |
| Eptinezumab | 69.5 |
| Galcanezumab | 61.8 |
| Atogepant | 56.2 |
| Amitriptyline | 54.7 |
| Topiramate | 52.7 |
| Candesartan | 47.9 |
| Erenumab | 39.3 |
| Propranolol | 31.1 |
| Rimegepant | 17.8 |
| Placebo | 2.9 |

SUCRA values vary from 0 to 1, whereas 1 means the treatment might be the best and 0 the worst.

**Table S5:** SUCRA of frequency of any adverse event

| **Treatment** | **SUCRA, n%** |
| --- | --- |
| Fremanezumab | 17.2 |
| Placebo | 22.6 |
| Erenumab | 25.5 |
| Rimegepant | 27.4 |
| Eptinezumab | 41.2 |
| Memantine | 46.4 |
| Sodium valproate | 47.9 |
| Galcanezumab | 48.9 |
| Atogepant | 65.3 |
| Propranolol | 68.3 |
| Topiramate | 87.4 |
| Amitriptyline | 100 |

SUCRA values vary from 0 to 1, whereas 1 means the most adverse events.

**Table S6:** Design-by-treatment interaction model for inconsistency of network meta-analysis

| **design-by-treatment inconsistency** | **chi2** | **Prob>chi2** |
| --- | --- | --- |
| change from baseline in migraine days | 5.18 | 0.26 |
| 50% response rate | 3.97 | 0.25 |
| adverse events leading to discontinuation | 2.22 | 0.69 |
| frequency of any adverse event | 2.49 | 0.28 |

**Table S7:** Significant loop-specific inconsistencies of network meta-analysis

| **loop inconsistency** | **Ratio of odds ratios** | **95% confidence interval** | **Tau^2^** | **P** |
| --- | --- | --- | --- | --- |
| **Change from baseline in migraine days** |  |  |  |  |
| Candesartan-Propranolol-placebo | 1.54 | (1.00, 2.54) | 0.00 | 0.09 |
| Propranolol-Topiramate-placebo | 1.18 | (1.00, 2.00) | 0.02 | 0.52 |
| **50% response rate** |  |  |  |  |
| Propranolol-Topiramate-placebo | 1.82 | (1.00, 8.18) | 0.25 | 0.43 |
| Candesartan-Propranolol-placebo | 1.35 | (1.00, 4.46) | 0.00 | 0.61 |
| **Adverse events leading to discontinuation** |  |  |  |  |
| Propranolol-Topiramate-placebo | 1.73 | (1.00, 4.24) | 0.000 | 0.22 |
| Candesartan-Propranolol-placebo | 1.37 | (1.00, 15.00) | 0.000 | 0.79 |
| **Frequency of any adverse event** |  |  |  |  |
| Propranolol-Topiramate-placebo | 1.36 | (1.00, 3.97) | 0.05 | 0.56 |
|  |  |  |  |  |

**Table S8:** Significant side-splitting inconsistencies of network meta-analysis

| **Side** | **Direct**  **Coefficient** | **SE** | **Indirect**  **Coefficient** | **SE** | **Difference**  **Coefficient** | **SE** | **p>z** | **Tau** | **Treatments used** |
| --- | --- | --- | --- | --- | --- | --- | --- | --- | --- |
| **Change from baseline in migraine days** |  |  |  |  |  |  |  |  |  |
| A VS. L | 0.41 | 0.22 | 0.44 | 17.41 | -0.03 | 17.41 | 1.00 | 0.11 | A: Amitriptyline |
| B VS.L | 0.41 | 0.11 | 0.41 | 141.71 | 0.00 | 141.71 | 1.00 | 0.11 | B: Atogepant |
| C VS. I | -0.21 | 0.21 | 0.24 | 0.24 | -0.45 | 0.33 | 0.17 | 0.11 | C: Candesartan |
| C VS. L | 0.44 | 0.15 | -0.39 | 0.45 | 0.83 | 0.47 | 0.08 | 0.10 | D: Eptinezumab |
| D VS. L | 0.39 | 0.07 | 0.42 | 99.96 | -0.03 | 99.96 | 1.00 | 0.11 | E: Erenumab |
| E VS. L | 0.38 | 0.06 | 0.44 | 82.27 | -0.06 | 82.27 | 1.00 | 0.11 | F: Fremanezumab |
| F VS. L | 0.56 | 0.08 | 0.26 | 100.79 | 0.30 | 100.79 | 1.00 | 0.11 | G: Galcanezumab |
| G VS. L | 0.46 | 0.07 | 0.36 | 89.49 | 0.10 | 89.49 | 1.00 | 0.11 | H: Memantine |
| H VS. L | 0.83 | 0.22 | -0.02 | 141.54 | 0.85 | 141.54 | 1.00 | 0.11 | I: Propranolol |
| I VS. K | -0.21 | 0.18 | 0.11 | 0.17 | -0.32 | 0.25 | 0.20 | 0.11 | J: Rimegepant |
| I VS. L | 0.37 | 0.12 | 0.39 | 0.31 | -0.02 | 0.33 | 0.95 | 0.11 | K: Topiramate |
| J VS. L | 0.18 | 0.13 | 0.64 | 200.07 | -0.46 | 200.07 | 1.00 | 0.11 | L: Placebo |
| K VS. L | 0.38 | 0.08 | 1.30 | 0.42 | -0.92 | 0.43 | 0.03 | 0.10 |  |
| A VS. L | 0.41 | 0.22 | 0.44 | 17.41 | -0.03 | 17.41 | 1.00 | 0.11 |  |
| **50% response rate** |  |  |  |  |  |  |  |  |  |
| A VS. L | -1.72 | 0.74 | -0.90 | 18.91 | -0.82 | 18.93 | 0.97 | 0.34 | A: Memantine |
| B VS. L | -0.92 | 0.54 | -2.53 | 199.88 | 1.61 | 199.88 | 0.99 | 0.34 | B: Amitriptyline |
| C VS. L | -0.92 | 0.29 | -2.52 | 141.44 | 1.59 | 141.44 | 0.99 | 0.34 | C: Atogepant |
| D VS. L | -1.07 | 0.20 | -2.37 | 100.02 | 1.30 | 100.02 | 0.99 | 0.34 | D: Eptinezumab |
| E VS. L | -0.73 | 0.17 | -2.71 | 81.69 | 1.98 | 81.69 | 0.98 | 0.34 | E: Erenumab |
| F VS. L | -1.21 | 0.22 | -2.23 | 100.03 | 1.02 | 100.03 | 0.99 | 0.34 | F: Fremanezumab |
| G VS. L | -0.98 | 0.20 | -2.46 | 100.02 | 1.48 | 100.02 | 0.99 | 0.34 | G: Galcanezumab |
| H VS. I | -0.12 | 0.52 | -0.64 | 1.15 | 0.52 | 1.26 | 0.68 | 0.35 | H: Candesartan |
| H VS. L | -0.90 | 0.54 | -0.38 | 1.12 | -0.52 | 1.26 | 0.68 | 0.35 | I: Propranolol |
| I VS. K | -0.12 | 0.44 | 0.70 | 0.45 | -0.82 | 0.63 | 0.20 | 0.33 | J: Rimegepant |
| I VS. L | -0.52 | 0.29 | -1.33 | 0.97 | 0.80 | 1.02 | 0.43 | 0.35 | K: Topiramate |
| J VS. L | -0.31 | 0.37 | -3.13 | 199.86 | 2.83 | 199.86 | 0.99 | 0.34 | L: Placebo |
| K VS. L | -0.95 | 0.23 | 0.54 | 1.07 | -1.49 | 1.09 | 0.17 | 0.33 |  |
| **Adverse events leading to discontinuation** |  |  |  |  |  |  |  |  |  |
| A VS.K | 0.62 | 1.28 | -0.57 | 17.96 | 1.18 | 18.01 | 0.95 | 0.12 | A: Memantine |
| B VS.K | -0.61 | 0.45 | 1.84 | 141.45 | -2.45 | 141.45 | 0.99 | 0.12 | B: Atogepant |
| C VS.K | -0.79 | 0.43 | 2.02 | 100.04 | -2.81 | 100.04 | 0.98 | 0.12 | C: Eptinezumab |
| D VS.K | -0.42 | 0.52 | 1.65 | 81.69 | -2.07 | 81.70 | 0.98 | 0.12 | D: Erenumab |
| E VS.K | -0.30 | 0.45 | 1.52 | 100.03 | -1.82 | 100.03 | 0.99 | 0.12 | E: Fremanezumab |
| F VS.K | -0.45 | 0.40 | 1.68 | 89.49 | -2.14 | 89.49 | 0.98 | 0.12 | F: Galcanezumab |
| G VS.H | -0.07 | 0.85 | -0.73 | 1.10 | 0.66 | 1.39 | 0.64 | 0.13 | G: Candesartan |
| G VS.K | -1.17 | 0.83 | -0.04 | 1.73 | -1.13 | 2.09 | 0.59 | 0.13 | H: Propranolol |
| H VS.J | 0.35 | 0.28 | -0.50 | 0.53 | 0.85 | 0.59 | 0.15 | 0.00 | I: Rimegepant |
| H VS.K | -0.81 | 0.31 | 0.21 | 0.61 | -1.02 | 0.72 | 0.16 | 0.00 | J: Topiramate |
| I VS.K | -0.57 | 0.64 | 1.80 | 200.11 | -2.37 | 200.12 | 0.99 | 0.12 | K: Placebo |
| J VS.K | -0.72 | 0.17 | -1.47 | 1.46 | 0.75 | 1.48 | 0.61 | 0.14 |  |
| **Frequency of any**  **adverse event** |  |  |  |  |  |  |  |  |  |
| A VS.K | -0.21 | 0.57 | -0.25 | 18.89 | 0.04 | 18.90 | 1.00 | 0.12 | A: Memantine |
| B VS.K | -2.17 | 0.44 | 1.74 | 199.94 | -3.91 | 199.94 | 0.98 | 0.12 | B: Amitriptyline |
| C VS.K | -0.35 | 0.18 | -0.08 | 141.50 | -0.27 | 141.50 | 1.00 | 0.12 | C: Atogepant |
| D VS.K | -0.11 | 0.11 | -0.32 | 99.87 | 0.21 | 99.87 | 1.00 | 0.12 | D: Eptinezumab |
| E VS.K | -0.01 | 0.10 | -0.42 | 81.72 | 0.41 | 81.72 | 1.00 | 0.12 | E: Erenumab |
| F VS.K | 0.06 | 0.12 | -0.48 | 100.01 | 0.54 | 100.01 | 1.00 | 0.12 | F: Fremanezumab |
| G VS.K | -0.16 | 0.10 | -0.27 | 89.67 | 0.11 | 89.67 | 1.00 | 0.12 | G: Galcanezumab |
| H VS.J | 0.48 | 0.31 | 0.22 | 0.36 | 0.26 | 0.47 | 0.58 | 0.13 | H: Propranolol |
| H VS.K | -0.53 | 0.24 | 0.35 | 0.67 | -0.88 | 0.73 | 0.23 | 0.12 | I: Rimegepant |
| I VS.K | 0.00 | 0.20 | -0.42 | 200.75 | 0.42 | 200.75 | 1.00 | 0.12 | J: Topiramate |
| J VS.K | -0.80 | 0.17 | -0.47 | 0.85 | -0.33 | 0.88 | 0.70 | 0.13 | K: Placebo |

**Table S9.** League table of 50% response rate

| **Memantine** |  |  |  |  |  |  |  |  |  |  | **5.60 (1.33,23.50)** |
| --- | --- | --- | --- | --- | --- | --- | --- | --- | --- | --- | --- |
| 2.23 (0.37,13.39) | **Amitriptyline** |  |  |  |  |  |  |  |  |  | 2.50 (0.88,7.08) |
| 2.21 (0.47,10.48) | 0.99 (0.30,3.31) | **Atogepant** |  |  |  |  |  |  |  |  | **2.52 (1.45,4.39)** |
| 1.91 (0.43,8.53) | 0.86 (0.28,2.65) | 0.86 (0.43,1.72) | **Eptinezumab** |  |  |  |  |  |  |  | **2.93 (2.01,4.27)** |
| 2.68 (0.61,11.86) | 1.20 (0.40,3.67) | 1.21 (0.62,2.36) | 1.41 (0.84,2.36) | **Erenumab** |  |  |  |  |  |  | **2.07 (1.48,2.86)** |
| 1.66 (0.37,7.48) | 0.74 (0.24,2.33) | 0.75 (0.37,1.53) | 0.87 (0.49,1.55) | 0.62 (0.36,1.06) | **Fremanezumab** |  |  |  |  |  | **3.36 (2.23,5.05)** |
| 2.09 (0.47,9.32) | 0.94 (0.30,2.89) | 0.94 (0.47,1.88) | 1.09 (0.63,1.89) | 0.78 (0.47,1.30) | 1.26 (0.71,2.23) | **Galcanezumab** |  |  |  |  | **2.67 (1.84,3.86)** |
| 2.50 (0.45,13.95) | 1.12 (0.28,4.59) | 1.13 (0.38,3.36) | 1.31 (0.48,3.59) | 0.93 (0.35,2.50) | 1.51 (0.55,4.18) | 1.20 (0.44,3.27) | **Candesartan** | 1.13 (0.42,2.99) |  |  | 2.46 (0.87,6.85) |
| 3.07 (0.66,14.39) | 1.38 (0.42,4.53) | 1.39 (0.63,3.05) | 1.61 (0.83,3.14) | 1.14 (0.60,2.17) | 1.85 (0.93,3.68) | 1.47 (0.76,2.86) | 1.23 (0.49,3.05) | **Propranolol** |  | 1.12 (0.48,2.64) | 1.69 (0.97,2.96) |
| 4.09 (0.81,20.73) | 1.84 (0.51,6.67) | 1.85 (0.73,4.69) | 2.15 (0.94,4.93) | 1.52 (0.68,3.42) | 2.47 (1.06,5.76) | 1.96 (0.86,4.49) | 1.63 (0.50,5.33) | 1.33 (0.54,3.32) | **Rimegepant** |  | 1.36 (0.67,2.75) |
| 2.31 (0.51,10.52) | 1.04 (0.33,3.28) | 1.04 (0.50,2.16) | 1.21 (0.67,2.20) | 0.86 (0.49,1.51) | 1.39 (0.75,2.59) | 1.11 (0.61,2.00) | 0.92 (0.34,2.51) | 0.75 (0.40,1.41) | 0.56 (0.24,1.34) | **Topiramate** | **2.59 (1.65,4.08)** |
| **5.58 (1.31,23.69)** | 2.50 (0.87,7.22) | **2.52 (1.42,4.47)** | **2.93 (1.98,4.33)** | **2.08 (1.48,2.91)** | **3.36 (2.21,5.13)** | **2.67 (1.82,3.92)** | 2.23 (0.88,5.62) | **1.82 (1.06,3.12)** | 1.36 (0.65,2.84) | **2.41 (1.54,3.79)** | **Placebo** |

Pairwise (upper-right portion) and network (lower-left portion) meta-analysis results are presented as estimated effect sizes for 50% response rate. For the result, outcomes are expressed as odds ratios (OR) with 95% credible interval (CI) (OR of > 1 indicated that the treatment specified in the row got more improvement than that specified in the column), 0 < OR < 1, the opposite. For the network meta-analysis, OR of > 1 indicates that the treatment specified in the column got better improvement than that specified in the row, 0 < OR < 1, the opposite. 95% CI that did not contain one was considered to have a statistical difference. Bold results indicate statistical significance.

**Table S10.** League table of frequency of any adverse event

| **Memantine** | 1.43 (0.11,8.51) |  |  |  |  |  |  |  |  |  | 1.24 (0.40,3.78) |
| --- | --- | --- | --- | --- | --- | --- | --- | --- | --- | --- | --- |
| 1.14 (0.28,4.60) | **Sodium valproate** |  |  |  |  |  |  |  |  |  | NA |
| 0.87 (0.27,2.82) | 0.76 (0.12,4.72) | **Atogepant** |  |  |  |  |  |  |  |  | **1.42 (1.00,2.01)** |
| 1.11 (0.36,3.47) | 0.97 (0.16,5.88) | 1.27 (0.84,1.93) | **Eptinezumab** |  |  |  |  |  |  |  | 1.11 (0.90,1.39) |
| 1.23 (0.40,3.82) | 1.07 (0.18,6.48) | 1.41 (0.94,2.10) | 1.10 (0.83,1.48) | **Erenumab** |  |  |  |  |  |  | 1.01 (0.83,1.22) |
| 1.31 (0.42,4.11) | 1.15 (0.19,6.95) | 1.50 (0.97,2.31) | 1.18 (0.85,1.63) | 1.07 (0.78,1.46) | **Fremanezumab** |  |  |  |  |  | 0.95 (0.74,1.20) |
| 1.06 (0.34,3.28) | 0.92 (0.15,5.57) | 1.21 (0.81,1.81) | 0.95 (0.71,1.27) | 0.86 (0.65,1.13) | 0.81 (0.59,1.10) | **Galcanezumab** |  |  |  |  | 1.17 (0.97,1.43) |
| 1.23 (0.38,4.02) | 1.08 (0.17,6.70) | 1.41 (0.84,2.38) | 1.11 (0.71,1.73) | 1.00 (0.65,1.54) | 0.94 (0.60,1.48) | 1.17 (0.76,1.80) | **Rimegepant** |  | 0.63 (0.35,1.16) |  | 1.00 (0.68,1.48) |
| **0.14 (0.03,0.58)** | **0.12 (0.02,0.90)** | **0.16 (0.06,0.41)** | **0.13 (0.05,0.31)** | **0.12 (0.05,0.28)** | **0.11 (0.04,0.27)** | **0.13 (0.06,0.33)** | **0.11 (0.04,0.30)** | **Amitriptyline** |  |  | **8.74 (3.67,20.84)** |
| 0.82 (0.25,2.69) | 0.71 (0.11,4.47) | 0.93 (0.54,1.62) | 0.73 (0.46,1.18) | 0.66 (0.42,1.06) | 0.62 (0.38,1.02) | 0.77 (0.48,1.23) | 0.66 (0.37,1.17) | **5.76 (2.19,15.14)** | **Propranolol*** |  | **1.70 (1.07,2.71)** |
| 0.57 (0.18,1.81) | 0.50 (0.08,3.04) | 0.65 (0.40,1.04) | 0.51 (0.35,0.75) | 0.46 (0.32,0.67) | 0.43 (0.29,0.65) | 0.54 (0.37,0.78) | 0.46 (0.28,0.76) | **4.00 (1.58,10.09)** | 0.69 (0.44,1.10) | **Topiramate** | **2.22 (1.60,3.10)** |
| 1.24 (0.40,3.78) | 1.08 (0.18,6.46) | **1.42 (1.00,2.02)** | 1.11 (0.90,1.38) | 1.01 (0.83,1.22) | 0.95 (0.74,1.21) | 1.17 (0.96,1.43) | 1.00 (0.68,1.48) | **8.74 (3.67,20.84)** | 1.52 (0.99,2.32) | **2.19 (1.59,3.02)** | **Placebo** |

Pairwise (upper-right portion) and network (lower-left portion) meta-analysis results are presented as estimated effect sizes. For the result, outcomes are expressed as odds ratios (OR) with 95% credible interval (CI). For the pairwise meta-analyses, OR < 1 indicates the treatment specified in the row had a fewer adverse event than that specified in the column. For the network meta-analysis, OR < 1 indicates the treatment specified in the column had a fewer adverse event than that specified in the row. 95% CI that did not contain one was considered to have a statistical difference. Bold results indicated statistical significance. Marked with * indicated a significant difference between direct and mixed compari

**Table S11.** Subgroup analysis of changes in frequency of migraine days

| **Amitriptyline** |  |  |  |  |  |  |  |
| --- | --- | --- | --- | --- | --- | --- | --- |
| -0.00 (-0.45,0.45) | **Atogepant** |  |  |  |  |  |  |
| -0.05 (-0.54,0.43) | -0.05 (-0.39,0.28) | **Candesartan** |  |  |  |  |  |
| 0.42 (-0.15,1.00) | 0.43 (-0.03,0.88) | 0.48 (-0.02,0.97) | **Memantine** |  |  |  |  |
| -0.04 (-0.49,0.41) | -0.04 (-0.33,0.25) | 0.01 (-0.29,0.32) | -0.47 (-0.93,-0.00) | **Propranolol** |  |  |  |
| -0.23 (-0.69,0.23) | -0.23 (-0.53,0.07) | -0.18 (-0.53,0.18) | **-0.66 (-1.13,-0.18)** | -0.19 (-0.50,0.12) | **Rimegepant** |  |  |
| 0.00 (-0.43,0.43) | 0.00 (-0.24,0.25) | 0.06 (-0.25,0.36) | -0.42 (-0.86,0.02) | 0.04 (-0.18,0.27) | 0.23 (-0.04,0.50) | **Topiramate** |  |
| **-0.41 (-0.81,-0.01)** | **-0.41 (-0.60,-0.21)** | **-0.35 (-0.63,-0.08)** | **-0.83 (-1.25,-0.42)** | **-0.37 (-0.58,-0.16)** | -0.18 (-0.40,0.05) | **-0.41 (-0.55,-0.26)** | **Placebo** |

For the result, outcomes are expressed as SMD and 95% confidence interval. SMD < 0 indicates the treatment specified in the column showed better improvement in migraine attack frequency than that specified in the row. Bold results indicated statistical significance.

**Table S12.** Subgroup analysis of withdrawal due to adverse events

| **Memantine** |  |  |  |  |  |  |
| --- | --- | --- | --- | --- | --- | --- |
| 0.29 (0.02,4.20) | **Atogepant** |  |  |  |  |  |
| 0.22 (0.01,3.72) | 0.75 (0.15,3.62) | **Candesartan** |  |  |  |  |
| 0.30 (0.02,3.93) | 1.03 (0.37,2.88) | 1.38 (0.37,5.11) | **Propranolol** |  |  |  |
| 0.30 (0.02,5.05) | 1.04 (0.22,4.81) | 1.39 (0.23,8.58) | 1.01 (0.26,3.98) | **Rimegepant** |  |  |
| 0.26 (0.02,3.26) | 0.88 (0.35,2.26) | 1.18 (0.31,4.50) | 0.86 (0.50,1.48) | 0.85 (0.23,3.11) | **Topiramate** |  |
| 0.54 (0.04,6.65) | 1.84 (0.76,4.43) | 2.47 (0.66,9.18) | **1.79 (1.04,3.09)** | 1.77 (0.50,6.21) | **2.08 (1.50,2.89)** | **Placebo** |

For the result, outcomes are expressed as odds ratios (OR) with 95% credible interval (CI). OR < 1 indicates the treatment specified in the column had a lower dropout rate than that specified in the row. 95% CI that did not contain one was considered to have a statistical difference. Bold results indicated statistical significanc

**Table S13.** Sensitivity analyses of changes in frequency of migraine days

| **Memantine** |  |  |  |  |  |  |  |  |  |  |  |  |
| --- | --- | --- | --- | --- | --- | --- | --- | --- | --- | --- | --- | --- |
| -0.43 (-1.03,0.18) | **Amitriptyline** |  |  |  |  |  |  |  |  |  |  |  |
| -0.43 (-0.91,0.05) | -0.00 (-0.48,0.48) | **Atogepant** |  |  |  |  |  |  |  |  |  |  |
| -0.42 (-1.02,0.19) | 0.01 (-0.60,0.61) | 0.01 (-0.48,0.49) | **Candesartan** |  |  |  |  |  |  |  |  |  |
| -0.44 (-0.89,0.01) | -0.02 (-0.47,0.43) | -0.01 (-0.28,0.25) | -0.02 (-0.48,0.43) | **Eptinezumab** |  |  |  |  |  |  |  |  |
| **-0.45 (-0.90,-0.01)** | -0.03 (-0.47,0.41) | -0.03 (-0.28,0.22) | -0.04 (-0.48,0.41) | -0.01 (-0.20,0.18) | **Erenumab** |  |  |  |  |  |  |  |
| -0.28 (-0.73,0.17) | 0.15 (-0.30,0.60) | 0.15 (-0.12,0.42) | 0.14 (-0.32,0.60) | 0.16 (-0.05,0.37) | 0.18 (-0.02,0.37) | **Fremanezumab** |  |  |  |  |  |  |
| -0.38 (-0.82,0.07) | 0.05 (-0.40,0.49) | 0.05 (-0.21,0.30) | 0.04 (-0.41,0.49) | 0.06 (-0.13,0.26) | 0.08 (-0.10,0.25) | -0.10 (-0.30,0.10) | **Galcanezumab** |  |  |  |  |  |
| -0.34 (-0.96,0.27) | 0.08 (-0.53,0.70) | 0.08 (-0.41,0.58) | 0.08 (-0.54,0.69) | 0.10 (-0.37,0.56) | 0.11 (-0.35,0.57) | -0.07 (-0.53,0.40) | 0.03 (-0.43,0.49) | **Propranolol** |  |  |  |  |
| **-0.66 (-1.16,-0.16)** | -0.23 (-0.73,0.27) | -0.23 (-0.57,0.11) | -0.24 (-0.74,0.27) | -0.21 (-0.52,0.09) | -0.20 (-0.49,0.09) | **-0.38 (-0.68,-0.07)** | -0.28 (-0.57,0.02) | -0.31 (-0.83,0.20) | **Rimegepant** |  |  |  |
| -0.32 (-0.90,0.25) | 0.10 (-0.48,0.68) | 0.10 (-0.34,0.55) | 0.10 (-0.49,0.68) | 0.12 (-0.30,0.54) | 0.13 (-0.28,0.54) | -0.05 (-0.47,0.38) | 0.05 (-0.36,0.47) | 0.02 (-0.57,0.61) | 0.33 (-0.14,0.81) | **Topiramate** |  |  |
| 0.18 (-0.61,0.96) | 0.60 (-0.39,1.59) | 0.60 (-0.32,1.53) | 0.60 (-0.40,1.59) | 0.62 (-0.29,1.52) | 0.63 (-0.27,1.53) | 0.45 (-0.45,1.36) | 0.55 (-0.35,1.46) | 0.52 (-0.48,1.52) | 0.83 (-0.10,1.77) | 0.50 (-0.48,1.48) | **Sodium valproate** |  |
| **-0.83 (-1.26,-0.41)** | -0.41 (-0.83,0.02) | **-0.41 (-0.63,-0.19)** | -0.41 (-0.85,0.02) | **-0.39 (-0.54,-0.25)** | **-0.38 (-0.50,-0.26)** | **-0.56 (-0.71,-0.40)** | **-0.46 (-0.58,-0.33)** | **-0.49 (-0.93,-0.05)** | -0.18 (-0.44,0.09) | **-0.51 (-0.90,-0.12)** | **-1.01 (-1.91,-0.12)** | **Placebo** |

For the result, outcomes are expressed as SMD and 95% confidence interval. SMD < 0 indicates the treatment specified in the column showed better improvement in migraine attack frequency than that specified in the row. Bold results indicated statistical significance.

**Table S14.** Sensitivity analyses of withdrawal due to adverse events

| **Atogepant** |  |  |  |  |  |  |  |  |  |  |
| --- | --- | --- | --- | --- | --- | --- | --- | --- | --- | --- |
| 0.84 (0.25,2.79) | **Eptinezumab** |  |  |  |  |  |  |  |  |  |
| 1.20 (0.32,4.55) | 1.43 (0.39,5.31) | **Erenumab** |  |  |  |  |  |  |  |  |
| 1.37 (0.40,4.66) | 1.63 (0.49,5.42) | 1.14 (0.30,4.32) | **Fremanezumab** |  |  |  |  |  |  |  |
| 1.17 (0.37,3.73) | 1.39 (0.45,4.33) | 0.97 (0.27,3.48) | 0.86 (0.27,2.73) | **Galcanezumab** |  |  |  |  |  |  |
| 3.41 (0.24,48.41) | 4.05 (0.29,56.98) | 2.83 (0.19,42.39) | 2.49 (0.17,35.39) | 2.91 (0.21,40.15) | **Memantine** |  |  |  |  |  |
| 1.04 (0.23,4.69) | 1.23 (0.28,5.48) | 0.86 (0.17,4.26) | 0.76 (0.17,3.43) | 0.89 (0.21,3.80) | 0.30 (0.02,4.99) | **Rimegepant** |  |  |  |  |
| 0.59 (0.05,6.84) | 0.70 (0.06,8.05) | 0.49 (0.04,6.02) | 0.43 (0.04,5.00) | 0.50 (0.04,5.66) | 0.17 (0.01,5.18) | 0.57 (0.04,7.70) | **Candesartan** |  |  |  |
| 1.83 (0.77,4.35) | 2.18 (0.95,5.01) | 1.52 (0.55,4.20) | 1.34 (0.56,3.19) | 1.57 (0.72,3.38) | 0.54 (0.04,6.62) | 1.77 (0.51,6.10) | 3.11 (0.31,30.84) | **Placebo** |  |  |
| 0.88 (0.13,6.16) | 1.05 (0.15,7.22) | 0.73 (0.10,5.50) | 0.64 (0.09,4.51) | 0.75 (0.11,5.06) | 0.26 (0.01,5.48) | 0.85 (0.10,7.20) | 1.49 (0.08,26.63) | 0.48 (0.08,2.74) | **Propranolol** |  |
| 0.82 (0.26,2.59) | 0.97 (0.31,3.01) | 0.68 (0.19,2.41) | 0.60 (0.19,1.90) | 0.70 (0.24,2.06) | 0.24 (0.02,3.30) | 0.79 (0.18,3.37) | 1.39 (0.12,15.54) | **0.45 (0.21,0.96)** | 0.93 (0.14,6.21) | **Topiramate** |

For the result, outcomes are expressed as SMD and 95% confidence interval. SMD < 0 indicates the treatment specified in the column showed better improvement in migraine attack frequency than that specified in the row. Bold results indicated statistical significance.

**Figure S1.** Overview of risk of bias


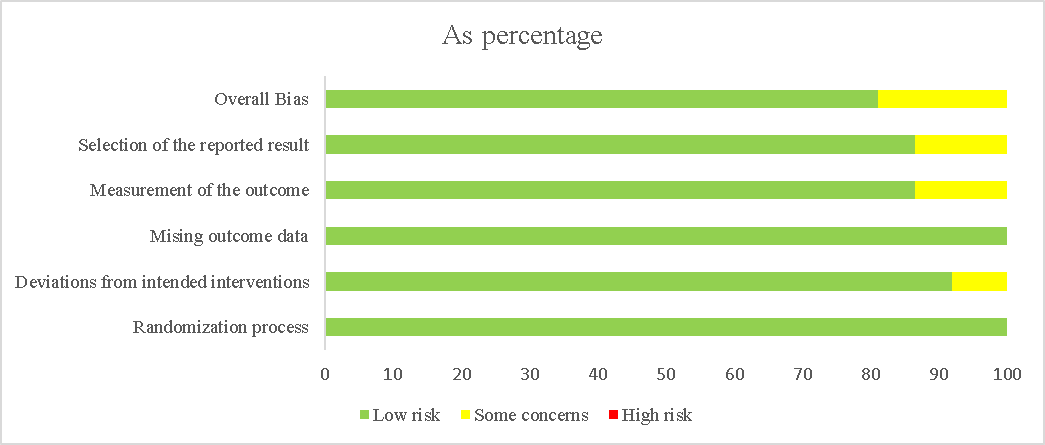


**Figure S2**. Detailed risk of bias in each study


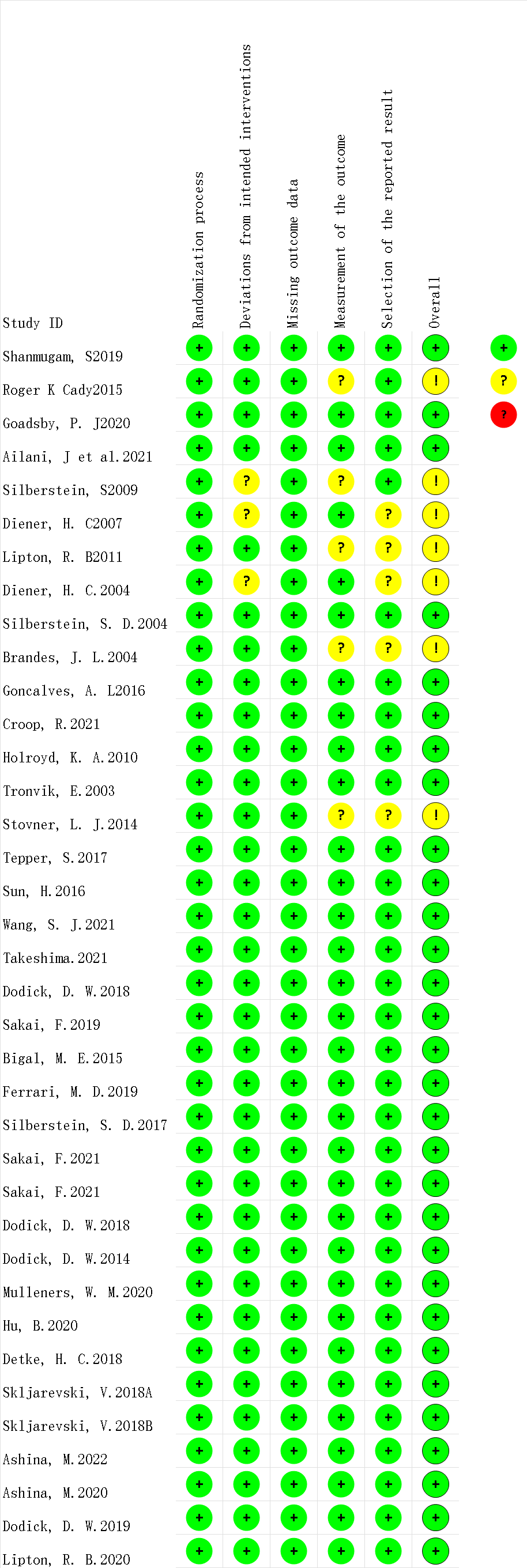


**Figure S3.** Funnel plot and Egger-value results of all studies included for all endpoints


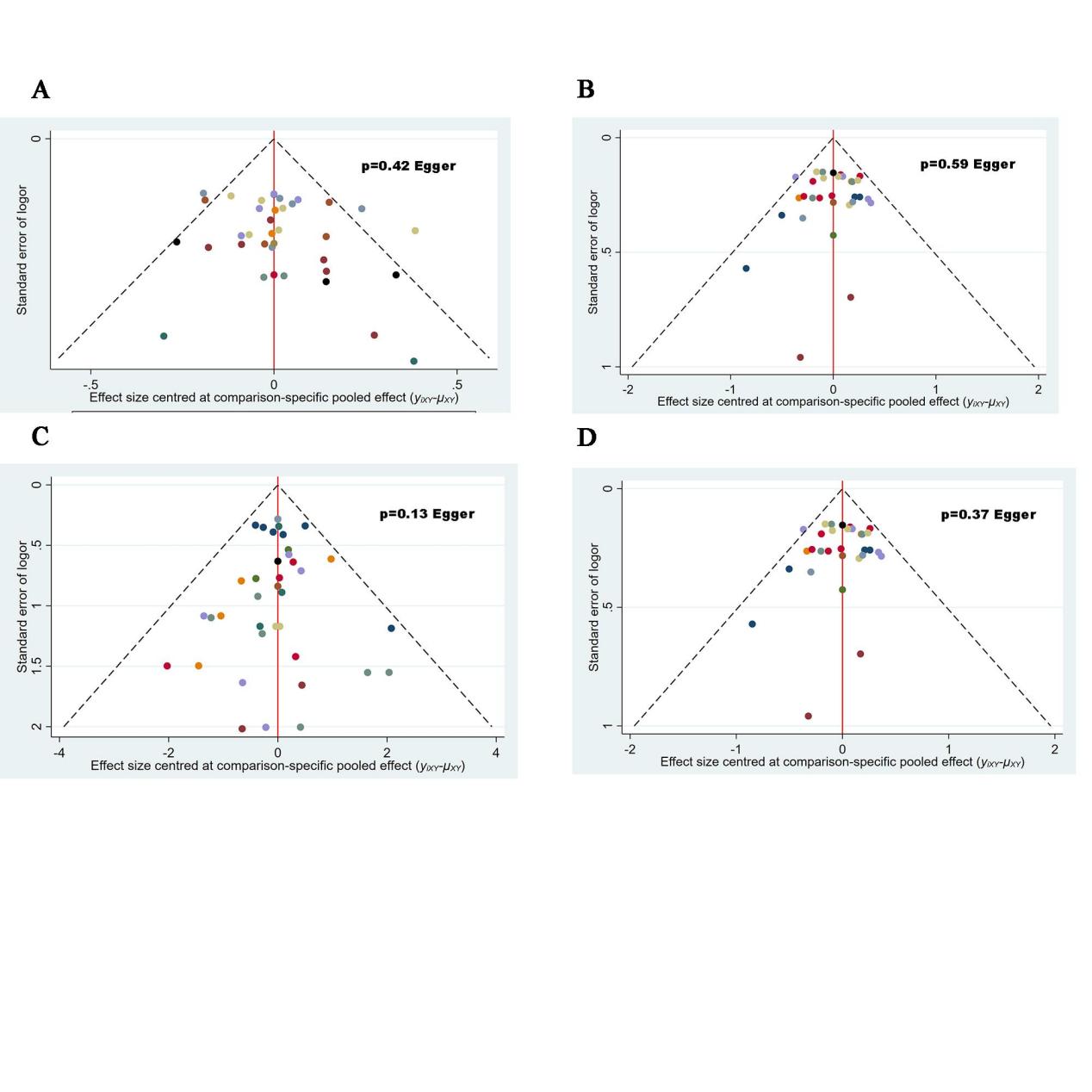


A, changes in frequency of migraine days. B, 50% the response rate. C, withdrawal due to adverse events. D, any adverse events within post-dose

**References:**

1. Guyatt, G., A. D. Oxman, E. A. Akl, R. Kunz, G. Vist, J. Brozek, S. Norris, Y. Falck-Ytter, P. Glasziou, H. Debeer, R. Jaeschke, D. Rind, J. Meerpohl, P. Dahm, and H. J. Schunemann (2011) GRADE guidelines: 1. Introduction-GRADE evidence profiles and summary of findings tables. J Clin Epidemiol. 64(4): 383-94.

2. Guyatt, G. H., A. D. Oxman, G. E. Vist, R. Kunz, Y. Falck-Ytter, P. Alonso-Coello, and H. J. Schunemann (2008) GRADE: an emerging consensus on rating quality of evidence and strength of recommendations. BMJ. 336(7650): 924-6.

3. Nikolakopoulou, A., Jpt Higgins, T. Papakonstantinou, A. Chaimani, Giovane C. Del, M. Egger, and G. Salanti (2020) CINeMA: An approach for assessing confidence in the results of a network meta-analysis. PLoS Med. 17(4): e1003082.

4. Sterne, Jac, J. Savovic, M. J. Page, R. G. Elbers, N. S. Blencowe, I. Boutron, C. J. Cates, H. Y. Cheng, M. S. Corbett, S. M. Eldridge, J. R. Emberson, M. A. Hernan, S. Hopewell, A. Hrobjartsson, D. R. Junqueira, P. Juni, J. J. Kirkham, T. Lasserson, T. Li, A. Mcaleenan, B. C. Reeves, S. Shepperd, I. Shrier, L. A. Stewart, K. Tilling, I. R. White, P. F. Whiting, and Jpt Higgins (2019) RoB 2: a revised tool for assessing risk of bias in randomised trials. BMJ. 366: l4898.

5. Papakonstantinou, T., A. Nikolakopoulou, Jpt Higgins, M. Egger, and G. Salanti (2020) CINeMA: Software for semiautomated assessment of the confidence in the results of network meta-analysis. Campbell Syst Rev. 16(1): e1080.

6. Locher, C., J. Kossowsky, H. Koechlin, T. L. Lam, J. Barthel, C. B. Berde, J. Gaab, G. Schwarzer, K. Linde, and K. Meissner (2020) Efficacy, Safety, and Acceptability of Pharmacologic Treatments for Pediatric Migraine Prophylaxis: A Systematic Review and Network Meta-analysis. JAMA Pediatr. 174(4): 341-349.

7. Koechlin, H., J. Kossowsky, T. L. Lam, J. Barthel, J. Gaab, C. B. Berde, G. Schwarzer, K. Linde, K. Meissner, and C. Locher (2021) Nonpharmacological Interventions for Pediatric Migraine: A Network Meta-analysis. Pediatrics. 147(4).

8. Noruzzadeh, R., A. Modabbernia, V. Aghamollaii, M. Ghaffarpour, M. H. Harirchian, S. Salahi, N. Nikbakht, N. Noruzi, and A. Tafakhori (2016) Memantine for Prophylactic Treatment of Migraine Without Aura: A Randomized Double-Blind Placebo-Controlled Study. Headache. 56(1): 95-103.

9. Shanmugam, S., K. Karunaikadal, S. Varadarajan, and M. Krishnan (2019) Memantine Ameliorates Migraine Headache. Ann Indian Acad Neurol. 22(3): 286-290.

10. Goadsby, P. J., D. W. Dodick, J. Ailani, J. M. Trugman, M. Finnegan, K. Lu, and A. Szegedi (2020) Safety, tolerability, and efficacy of orally administered atogepant for the prevention of episodic migraine in adults: a double-blind, randomised phase 2b/3 trial. Lancet Neurol. 19(9): 727-737.

11. Ailani, J., R. B. Lipton, P. J. Goadsby, H. Guo, R. Miceli, L. Severt, M. Finnegan, and J. M. Trugman (2021) Atogepant for the Preventive Treatment of Migraine. N Engl J Med. 385(8): 695-706.

12. Silberstein, S., R. Lipton, D. Dodick, F. Freitag, N. Mathew, J. Brandes, M. Bigal, S. Ascher, J. Morein, P. Wright, S. Greenberg, and J. Hulihan (2009) Topiramate treatment of chronic migraine: a randomized, placebo-controlled trial of quality of life and other efficacy measures. Headache. 49(8): 1153-62.

13. Diener, H. C., G. Bussone, J. C. Van Oene, M. Lahaye, S. Schwalen, and P. J. Goadsby (2007) Topiramate reduces headache days in chronic migraine: a randomized, double-blind, placebo-controlled study. Cephalalgia. 27(7): 814-23.

14. Lipton, R. B., S. Silberstein, D. Dodick, R. Cady, F. Freitag, N. Mathew, D. M. Biondi, S. Ascher, W. H. Olson, and J. Hulihan (2011) Topiramate intervention to prevent transformation of episodic migraine: the topiramate INTREPID study. Cephalalgia. 31(1): 18-30.

15. Diener, H. C., P. Tfelt-Hansen, C. Dahlof, M. J. Lainez, G. Sandrini, S. J. Wang, W. Neto, U. Vijapurkar, A. Doyle, and D. Jacobs (2004) Topiramate in migraine prophylaxis--results from a placebo-controlled trial with propranolol as an active control. J Neurol. 251(8): 943-50.

16. Silberstein, S. D., W. Neto, J. Schmitt, and D. Jacobs (2004) Topiramate in migraine prevention: results of a large controlled trial. Arch Neurol. 61(4): 490-5.

17. Brandes, J. L., J. R. Saper, M. Diamond, J. R. Couch, D. W. Lewis, J. Schmitt, W. Neto, S. Schwabe, and D. Jacobs (2004) Topiramate for migraine prevention: a randomized controlled trial. JAMA. 291(8): 965-73.

18. Goncalves, A. L., Ferreira A. Martini, R. T. Ribeiro, E. Zukerman, J. Cipolla-Neto, and M. F. Peres (2016) Randomised clinical trial comparing melatonin 3 mg, amitriptyline 25 mg and placebo for migraine prevention. J Neurol Neurosurg Psychiatry. 87(10): 1127-32.

19. Croop, R., R. B. Lipton, D. Kudrow, D. A. Stock, L. Kamen, C. M. Conway, E. G. Stock, V. Coric, and P. J. Goadsby (2021) Oral rimegepant for preventive treatment of migraine: a phase 2/3, randomised, double-blind, placebo-controlled trial. Lancet. 397(10268): 51-60.

20. Holroyd, K. A., C. K. Cottrell, F. J. O'Donnell, G. E. Cordingley, J. B. Drew, B. W. Carlson, and L. Himawan (2010) Effect of preventive (beta blocker) treatment, behavioural migraine management, or their combination on outcomes of optimised acute treatment in frequent migraine: randomised controlled trial. BMJ. 341: c4871.

21. Tronvik, E., L. J. Stovner, G. Helde, T. Sand, and G. Bovim (2003) Prophylactic treatment of migraine with an angiotensin II receptor blocker: a randomized controlled trial. JAMA. 289(1): 65-9.

22. Stovner, L. J., M. Linde, G. B. Gravdahl, E. Tronvik, A. H. Aamodt, T. Sand, and K. Hagen (2014) A comparative study of candesartan versus propranolol for migraine prophylaxis: A randomised, triple-blind, placebo-controlled, double cross-over study. Cephalalgia. 34(7): 523-32.

23. Tepper, S., M. Ashina, U. Reuter, J. L. Brandes, D. Dole啪il, S. Silberstein, P. Winner, D. Leonardi, D. Mikol, and R. Lenz (2017) Safety and efficacy of erenumab for preventive treatment of chronic migraine: a randomised, double-blind, placebo-controlled phase 2 trial. Lancet Neurol. 16(6): 425-434.

24. Sun, H., D. W. Dodick, S. Silberstein, P. J. Goadsby, U. Reuter, M. Ashina, J. Saper, R. Cady, Y. Chon, J. Dietrich, and R. Lenz (2016) Safety and efficacy of AMG 334 for prevention of episodic migraine: a randomised, double-blind, placebo-controlled, phase 2 trial. Lancet Neurol. 15(4): 382-90.

25. Wang, S. J., Aa Jr Roxas, B. Saravia, B. K. Kim, D. Chowdhury, N. Riachi, M. S. Tai, S. Tanprawate, T. T. Ngoc, Y. J. Zhao, D. D. Mikol, S. Pandhi, S. Wen, S. Mondal, N. Tenenbaum, and P. Hours-Zesiger (2021) Randomised, controlled trial of erenumab for the prevention of episodic migraine in patients from Asia, the Middle East, and Latin America: The EMPOwER study. Cephalalgia. 41(13): 1285-1297.

26. Takeshima, T., F. Sakai, K. Hirata, N. Imai, Y. Matsumori, R. Yoshida, C. Peng, S. Cheng, and D. D. Mikol (2021) Erenumab treatment for migraine prevention in Japanese patients: Efficacy and safety results from a Phase 3, randomized, double-blind, placebo-controlled study. Headache. 61(6): 927-935.

27. Dodick, D. W., M. Ashina, J. L. Brandes, D. Kudrow, M. Lanteri-Minet, V. Osipova, K. Palmer, H. Picard, D. D. Mikol, and R. A. Lenz (2018) ARISE: A Phase 3 randomized trial of erenumab for episodic migraine. Cephalalgia. 38(6): 1026-1037.

28. Sakai, F., T. Takeshima, Y. Tatsuoka, K. Hirata, R. Lenz, Y. Wang, S. Cheng, T. Hirama, and D. D. Mikol (2019) A Randomized Phase 2 Study of Erenumab for the Prevention of Episodic Migraine in Japanese Adults. Headache. 59(10): 1731-1742.

29. Bigal, M. E., D. W. Dodick, A. M. Rapoport, S. D. Silberstein, Y. Ma, R. Yang, P. S. Loupe, R. Burstein, L. C. Newman, and R. B. Lipton (2015) Safety, tolerability, and efficacy of TEV-48125 for preventive treatment of high-frequency episodic migraine: a multicentre, randomised, double-blind, placebo-controlled, phase 2b study. Lancet Neurol. 14(11): 1081-90.

30. Ferrari, M. D., H. C. Diener, X. Ning, M. Galic, J. M. Cohen, R. Yang, M. Mueller, A. H. Ahn, Y. C. Schwartz, M. Grozinski-Wolff, L. Janka, and M. Ashina (2019) Fremanezumab versus placebo for migraine prevention in patients with documented failure to up to four migraine preventive medication classes (FOCUS): a randomised, double-blind, placebo-controlled, phase 3b trial. Lancet. 394(10203): 1030-1040.

31. Silberstein, S. D., D. W. Dodick, M. E. Bigal, P. P. Yeung, P. J. Goadsby, T. Blankenbiller, M. Grozinski-Wolff, R. Yang, Y. Ma, and E. Aycardi (2017) Fremanezumab for the Preventive Treatment of Chronic Migraine. N Engl J Med. 377(22): 2113-2122.

32. Sakai, F., N. Suzuki, B. K. Kim, Y. Tatsuoka, N. Imai, X. Ning, M. Ishida, K. Nagano, K. Iba, H. Kondo, and N. Koga (2021) Efficacy and safety of fremanezumab for episodic migraine prevention: Multicenter, randomized, double-blind, placebo-controlled, parallel-group trial in Japanese and Korean patients. Headache. 61(7): 1102-1111.

33. Sakai, F., N. Suzuki, B. K. Kim, H. Igarashi, K. Hirata, T. Takeshima, X. Ning, T. Shima, M. Ishida, K. Iba, H. Kondo, and N. Koga (2021) Efficacy and safety of fremanezumab for chronic migraine prevention: Multicenter, randomized, double-blind, placebo-controlled, parallel-group trial in Japanese and Korean patients. Headache. 61(7): 1092-1101.

34. Dodick, D. W., S. D. Silberstein, M. E. Bigal, P. P. Yeung, P. J. Goadsby, T. Blankenbiller, M. Grozinski-Wolff, R. Yang, Y. Ma, and E. Aycardi (2018) Effect of Fremanezumab Compared With Placebo for Prevention of Episodic Migraine: A Randomized Clinical Trial. JAMA. 319(19): 1999-2008.

35. Dodick, D. W., P. J. Goadsby, E. L. Spierings, J. C. Scherer, S. P. Sweeney, and D. S. Grayzel (2014) Safety and efficacy of LY2951742, a monoclonal antibody to calcitonin gene-related peptide, for the prevention of migraine: a phase 2, randomised, double-blind, placebo-controlled study. Lancet Neurol. 13(9): 885-92.

36. Mulleners, W. M., B. K. Kim, Mja L谩inez, M. Lanteri-Minet, P. Pozo-Rosich, S. Wang, A. Tockhorn-Heidenreich, S. K. Aurora, R. M. Nichols, L. Yunes-Medina, and H. C. Detke (2020) Safety and efficacy of galcanezumab in patients for whom previous migraine preventive medication from two to four categories had failed (CONQUER): a multicentre, randomised, double-blind, placebo-controlled, phase 3b trial. Lancet Neurol. 19(10): 814-825.

37. Hu, B., G. Li, X. Li, S. Wu, T. Yu, X. Li, H. Zhao, Z. Jia, J. Zhuang, and S. Yu (2022) Galcanezumab in episodic migraine: the phase 3, randomized, double-blind, placebo-controlled PERSIST study. J Headache Pain. 23(1): 90.

38. Detke, H. C., P. J. Goadsby, S. Wang, D. I. Friedman, K. J. Selzler, and S. K. Aurora (2018) Galcanezumab in chronic migraine: The randomized, double-blind, placebo-controlled REGAIN study. Neurology. 91(24): e2211-e2221.

39. Skljarevski, V., M. Matharu, B. A. Millen, M. H. Ossipov, B. K. Kim, and J. Y. Yang (2018) Efficacy and safety of galcanezumab for the prevention of episodic migraine: Results of the EVOLVE-2 Phase 3 randomized controlled clinical trial. Cephalalgia. 38(8): 1442-1454.

40. Skljarevski, V., T. M. Oakes, Q. Zhang, M. B. Ferguson, J. Martinez, A. Camporeale, K. W. Johnson, Q. Shan, J. Carter, A. Schacht, P. J. Goadsby, and D. W. Dodick (2018) Effect of Different Doses of Galcanezumab vs Placebo for Episodic Migraine Prevention: A Randomized Clinical Trial. JAMA Neurol. 75(2): 187-193.

41. Ashina, M., M. Lanteri-Minet, P. Pozo-Rosich, A. Ettrup, C. L. Christoffersen, M. K. Josiassen, R. Phul, and B. Sperling (2022) Safety and efficacy of eptinezumab for migraine prevention in patients with two-to-four previous preventive treatment failures (DELIVER): a multi-arm, randomised, double-blind, placebo-controlled, phase 3b trial. Lancet Neurol. 21(7): 597-607.

42. Ashina, M., J. Saper, R. Cady, B. A. Schaeffler, D. M. Biondi, J. Hirman, S. Pederson, B. Allan, and J. Smith (2020) Eptinezumab in episodic migraine: A randomized, double-blind, placebo-controlled study (PROMISE-1). Cephalalgia. 40(3): 241-254.

43. Dodick, D. W., R. B. Lipton, S. Silberstein, P. J. Goadsby, D. Biondi, J. Hirman, R. Cady, and J. Smith (2019) Eptinezumab for prevention of chronic migraine: A randomized phase 2b clinical trial. Cephalalgia. 39(9): 1075-1085.

44. Lipton, R. B., P. J. Goadsby, J. Smith, B. A. Schaeffler, D. M. Biondi, J. Hirman, S. Pederson, B. Allan, and R. Cady (2020) Efficacy and safety of eptinezumab in patients with chronic migraine: PROMISE-2. Neurology. 94(13): e1365-e1377.

45. Vazquez-Guevara, Damaris, Alejandro Orozco-Narvaez, Hector G. Hernandez-Rodriguez, Francisco Rivas-Ruvalcaba, Juan Manuel Shiguetomi-Medina, and Ildefonso Rodriguez-Leyva (2023) Efficacy of memantine compared with sodium valproate as prophylactic treatment for migraine: a controlled randomized pilot study. Exploration of Neuroprotective Therapy. 3(2): 131-138.
